# Genome-wide expression atlas of tomato flower buds revealed the *SllncERF162*-*SlERF162* module associated with basal thermotolerance

**DOI:** 10.1093/hr/uhaf205

**Published:** 2025-07-31

**Authors:** Qinqin Yang, Xiaolin Geng, Hongwei Li, Yanqing Cong, Ming Zhou, Zhaoyang Zhou, Yune Cao, Yan Yan, Na Zhang, Yingfang Zhu, Tao Lin

**Affiliations:** College of Horticulture, China Agricultural University, Beijing 100193, China; College of Horticulture, China Agricultural University, Beijing 100193, China; College of Horticulture, China Agricultural University, Beijing 100193, China; College of Horticulture, China Agricultural University, Beijing 100193, China; Key Laboratory of Biology and Genetic Improvement of Horticultural Crops (North China), Ministry of Agriculture, Beijing Vegetable Research Center, Beijing Academy of Agriculture and Forestry Sciences, Beijing 100097, China; College of Horticulture, China Agricultural University, Beijing 100193, China; The School of Oenology and Horticulture, Ningxia University, Ningxia 750021, China; State Key Laboratory of Vegetable Biobreeding, Institute of Vegetables and Flowers, Chinese Academy of Agricultural Sciences, Beijing 100081, China; College of Horticulture, China Agricultural University, Beijing 100193, China; State Key Laboratory of Crop Stress Adaptation and Improvement, School of Life Sciences, Henan University, Kaifeng 475004, China; College of Horticulture, China Agricultural University, Beijing 100193, China

## Abstract

High temperatures impair pollen viability and reduce fruit set, ultimately affecting the yield of crops. Understanding the genetic components involved in the heat stress (HS) response is essential for developing climate-resilient crop varieties. However, the regulatory mechanisms governing HS responses during pollen development in tomato (*Solanum lycopersicum*) remain unexplored. In this study, we identified the microspore mother cell stage as the most heat-sensitive phase in tomato pollen development. Furthermore, we generated a comprehensive RNA expression profile of tomato flower buds under HS, encompassing 8051 mRNAs, 5738 lncRNAs, 62 circRNAs, and 24 miRNAs. Comparative analysis of these RNAs revealed three distinct response phases, early, late, and dual, and enabled the identification of coexpression modules comprising both coding and noncoding transcripts. Among these, *SlERF162* was identified as a key regulatory gene that promotes pollen thermotolerance. We further identified the lncRNA *TCONS_00023929* (designated *SllncERF162*) as a positive regulator of *SlERF162* expression. Both *SlERF162* and *SllncERF162* contributed to maintaining pollen viability under HS. Additional experiments demonstrated that the *SllncERF162–SlERF162* regulatory module enhances basal thermotolerance by directly targeting and activating the heat-responsive genes *SlHsfB1* and *SlsHSP*. Overall, this study provides a high-resolution expression atlas of RNAs under HS and uncovers a novel noncoding RNA-mediated regulatory network that promotes thermotolerance during tomato pollen development.

## Introduction

Global warming has significantly affected crop yield and quality, posing serious risks to food security and broader societal stability [1]. Temperature plays a critical role in determining plant growth and geographic distribution. Elevated temperatures commonly disrupt pollen development and cause abnormal floral growth, ultimately resulting in substantial fruit loss and decreased crop yield [2]. Tomato (*Solanum lycopersicum*), a major fruit crop, has developed complex mechanisms to cope with environmental stress. Heat stress (HS) during the early reproductive stage hinders flower bud differentiation, induces gametophyte abortion, and reduces blooming and fruit set [2, 3].

Over the past several decades, numerous genes associated with thermotolerance have been identified in plants [4, 5]. The core heat shock factor (HSF) network that mediates the HS response relies on the activities of *HsfA1a*, *HsfA2*, and *HsfB1* [6]. Among them, *SlHsfB1* plays a dual role as a coactivator and a repressor [7]. The HsfA1 family, recognized as a group of master regulators of thermotolerance, directly activates the expression of heat-responsive genes [8, 9]. In tomato, *HsfA2* contributes to pollen thermotolerance during microsporogenesis by functioning as a coactivator of *HsfA1a* [10]. Heat shock proteins (HSPs), including *HSP100*, *HSP90*, *HSP70*, and small HSPs (*sHSPs*), play essential roles in preserving protein stability under HS conditions [11]. In tomato, HSP70 represses the activity of *HsfA1*, while HSP90 influences the abundance of both *HsfA2* and *HsfB1* [6]. In addition, the AP2/ERF family, a large group of plant-specific transcription factors (TFs), modulates stress tolerance by activating HSFs [12–14]. For instance, DREB2A and DREB2C can activate the *HsfA3* promoter, thereby enhancing the expression of *HSP* genes and improving thermotolerance [15–17]. The *ERF95*-*ERF97* dimer has been shown to positively regulate the expression of *HsfA2* in *Arabidopsis* [18]. However, the genetic mechanisms by ethylene response factor (ERFs) regulate the HS response in tomato remain unknown.

**Figure 1:**
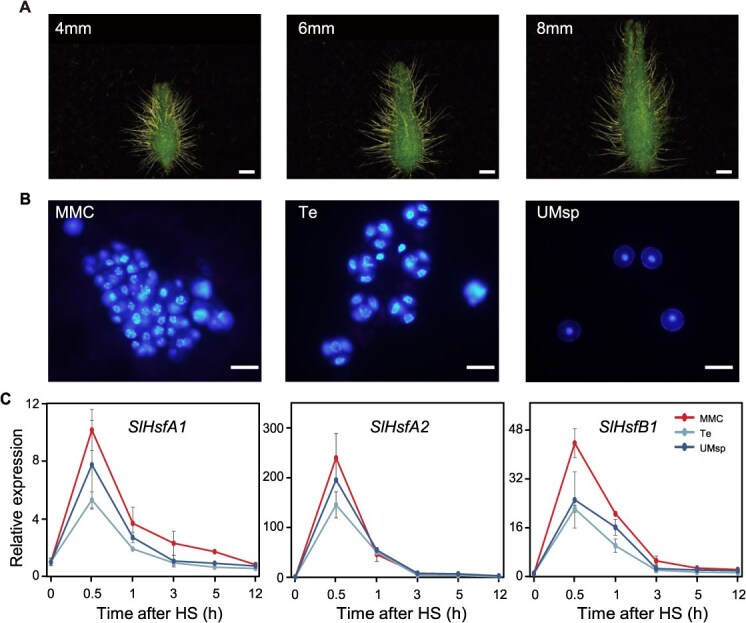
The developmental stages of tomato pollens and expression patterns of three *HSFs* under HS. (A) Morphological characteristics of tomato flower buds at different developmental stages. Bars = 1 mm. (B) DAPI-stained pollen nuclei corresponding to the stages of flower bud development in panel A. Bars = 20 μm. MMC, microspore mother cell. Te, tetrad. UMsp, uninucleate microspore. (C) Expression levels of three HSFs *SlHsfA1*, *SlHsfA2*, and *SlHsfB1* in tomato flower buds at different time points under HS. Values represent the mean ± SD (*n* = 3).

In addition to TFs, noncoding RNAs (ncRNAs) play crucial roles in HS response by modulating the activity of TFs or target genes. Several miRNAs have been identified in crop species, where they contribute to male sterility under HS conditions [19–22]. In *Arabidopsis*, miR156 isoforms sustain the expression of HS-responsive genes by targeting *SPL* genes [23]. In wheat, miR159 is involved in anther development and heat response through negative regulation of *TaGAMYB* expression [24]. Emerging evidence indicates that lncRNAs and circRNAs also participate in plant responses to abiotic stress [25]. For example, the lncRNA *FLINC* regulates ambient temperature-mediated flowering under HS in *Arabidopsis* [26], whereas *DANA2* enhances drought tolerance by recruiting *ERF84* to promote JMJ29-mediated histone demethylation [27]. In apple, *MdLNC499* facilitates the interaction between *MdWRKY1* and *MdERF109* to regulate early-stage light-induced anthocyanin accumulation [28], and *MdLNC610* promotes *MdACO1* expression, affecting anthocyanin biosynthesis under high-light treatment [29]. The circRNA *Vv-circATS1*, derived from a glycerol-3-P acyltransferase gene, enhances cold tolerance in grapes [30]. Additionally, both lncRNAs and circRNAs may act as molecular sponges by competing for binding to shared miRNA response elements, thereby inhibiting miRNAs activity [31]. In *Arabidopsis*, the lncRNA *IPS1* regulates phosphate homeostasis by sequestering miR399, which controls the expression of *PHO2* [32]. However, the contribution of ncRNAs to the regulation of TFs under HS in tomato remains unexplored.

Here, we present a comprehensive RNA expression profile of tomato flower buds under HS, by systematically identifying ncRNAs and characterizing their expression patterns. Based on their temporal expression, these RNAs are classified into three stages: early, late, and dual stages. Our coexpression network analysis revealed that *SllncERF162* is significantly induced under HS and enhances the expression of *SlERF162*. We further provide evidence to demonstrate that both *SllncERF162* and *SlERF162* contribute to the promotion of basal thermotolerance in tomato during pollen development.

## Results

### A potential sensitive developmental stage of tomato pollens under HS

Pollen development occurs within the anthers and begins in early-stage immature flower buds, which play a direct role in determining crop yield. To identify the potentially sensitive stage of pollen development in tomato, we examined cytological changes during flower bud maturation in tomato and analyzed the expression patterns of heat shock TFs under HS. DAPI staining revealed that pollens in 4-mm flower buds were at the microspore mother cell stage (MMC), those in 6-mm flower buds had transitioned to the tetrad stage (Te), and those in 8-mm flower buds had reached the uninucleate microspore stage (UMsp) (Fig. 1A and B). We conducted reverse transcription quantitative polymerase chain reaction (RT-qPCR) analysis to examine the expression patterns of three HSFs (*SlHsfA1*, *SlHsfA2*, *SlHsfB1*) during tomato flower bud development under HS. Notably, all three genes exhibited transient induction in response to HS, peaking at 0.5 h, followed by a gradual decline at subsequent time points (Fig. 1C). Interestingly, their expression levels were markedly higher in the MMC stage than in the Te stage and UMsp stage. These findings suggested that the MMC stage is particularly sensitive to HS during tomato pollen meiosis.

### Genome-wide expression profile of total RNAs in tomato flower buds under HS

To explore the genome-wide expression profile of HS-responsive RNAs, we conducted whole-transcriptome strand-specific RNA sequencing (ssRNA-seq) and small RNA sequencing (sRNA-seq) on tomato flower buds in the MMC stage. Samples were collected under control conditions (0 h) and following exposure to HS for 0.5, 1, 3, and 5 h. A total of 1.1 billion sequencing reads were generated, with each sample yielding at least 19.96 Gb of data suitable for identifying strand-specific RNAs (Table S1). After removing adaptor sequences and low-quality reads, the clean reads were aligned to the tomato reference genome SLT1.0 [33]. Between 98.88% and 99.46% of total reads passed quality control, and 92.80%–95.10% (average 93.68%) of these reads successfully mapped to the reference genome (Table S1). These results indicated that its suitability of the data for the identification of total RNAs in tomato flower buds.

A computational strategy for the genome-wide identification of total RNAs, including mRNAs, lncRNAs, circRNAs, was used for each sample (Fig. S1). First, the expression level of the predicated protein-coding genes (mRNAs) was normalized and measured as the fragments per kilobase per million reads (FPKM) by using the Stringtie software [34]. A total of 34 383 mRNAs were obtained in these samples. Second, to identify lncRNAs, transcripts shorter than 200 bp or lacking strand information were removed. Transcripts overlapping known protein-coding genes on the sense strand were excluded, and coding potential was assessed using the Coding Potential Calculator (CPC) and Coding-Non-Coding Index (CNCI). Transcripts with coding potential scores >0 were discarded. To further eliminate protein-coding sequences, transcripts containing known protein domains were excluded using PfamScan program [35]. This filtering process yielded 14 788 reliable lncRNAs, whose expression levels were also calculated as FPKM. Third, to comprehensively identify circRNAs, unmapped reads were extracted and screened for back-spliced junctions by using the find_circ tool [36]. A total of 7304 circRNAs were detected, and their abundance was normalized to the number of back-spliced reads per million clean reads (RPM). Forth, small RNA-Seq data were mapped to the tomato reference genome SLT1.0 [33] (Table S2). After removing rRNAs, tRNAs, sRNAs, and snRNAs based on the Rfam database (release 14.5) [37], a total of 409 miRNAs were identified, comprising 61 known and 348 putative novel sequences. An expression threshold of 0.1 was used to determine whether a given RNA species was considered expressed in a sample. Applying this stringent pipeline, we detected a total of 46 932 RNAs across the samples, including 25 171 mRNAs, 14 052 lncRNAs, 7304 circRNAs, and 405 miRNAs.

**Figure 2:**
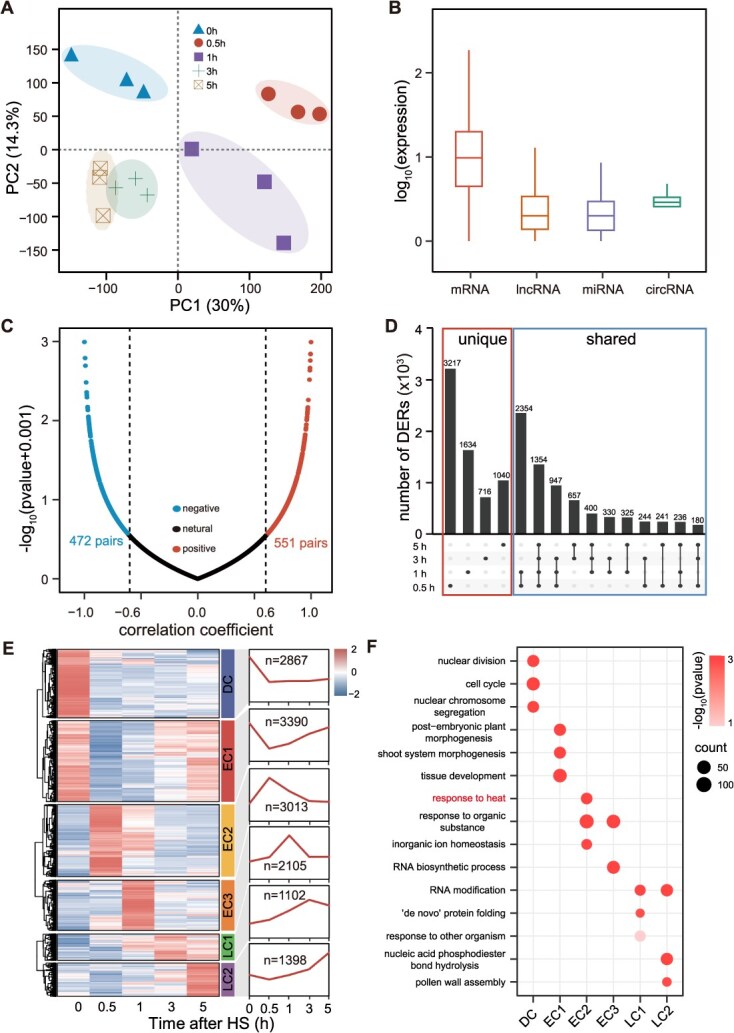
Overview of the genome-wide expression profile of total RNAs in tomato flower buds under HS. (A) PCA of genes expressed under control and HS. (B) Expression levels of mRNA, lncRNA, miRNA, and circRNA. (C) Scatter plots showing Pearson’s correlation coefficients and *P*-values between the expression levels of noncoding RNAs and their predicted target genes. (D) Number of DERs at each time point under HS. ‘Unique’ refers to RNAs differentially expressed at only one time point, and ‘shared’ refers to those RNAs differentially expressed at two or more time points. (E) Temporal expression patterns of DERs and their clustering under HS. The curve in right panel indicates the average expression level of RNAs within each cluster. DC, dual cluster. EC1, early cluster 1. EC2, early cluster 2. EC3, early cluster 3. LC1, late cluster 1. LC2, late cluster 2. (F) Functional enrichment analysis of each expression cluster.

To assess the consistency across biological replicates, we performed pairwise Spearman’s correlation analyses by using the expression values of all identified RNAs. The results revealed strong correlations among the three replicates at each time point (Fig. S2). We then conducted principal component analysis (PCA) across the five time points based on the 46 932 RNAs expressed in at least one sample. This analysis clearly distinguished between the control and HS samples, with samples from each time point forming distinct clusters (Fig. 2A). For downstream analyses, we calculated the average expression values across the replicates. Using the defined expression threshold, 45 078 RNAs were found to be expressed in at least one of the five time points. Of these, 28 501 RNAs including 21 670 mRNAs, 6240 lncRNAs, 292 circRNAs, and 299 miRNAs were expressed at all five time points (Fig. S3). The number of RNAs expressed at individual time points ranged from 33 865 at 0.5 h to 36 772 at 5 h (Fig. S4). We further categorized RNAs based on expression levels, between 9976 and 14 456 (17.54%–25.41% of total) showed high expressions (FPKM/RPM ≥ 10), 9151–10 182 RNAs (16.09%–17.90%) showed moderate expressions (2 ≤ FPKM/RPM < 10), 12 497–14 892 RNAs (21.97%–26.18%) showed low expressions (0.1 ≤ FPKM/RPM < 2), and 20 112–23 019 RNAs (35.26%–40.47) did not express at a given time point (Fig. S5). Collectively, these data provide a robust resource for exploring regulatory RNA networks in tomato flower buds under HS.

### Genomic characteristics of ncRNAs in tomato flower buds

We analyzed the genomic characteristics and expression profiles of the identified ncRNAs and compared them with those of mRNAs. The ncRNAs were found to be unevenly distributed across the 12 chromosomes (Fig. S6). Based on their genomic positions, the 14 788 lncRNAs were categorized into three types: 7940 intergenic lncRNAs, 5341 intronic or genic lncRNAs, and 1507 natural antisense long noncoding transcripts. Transcript lengths ranged from 204 to 44 718 nt, with a median of 1876 nt, which is longer than the median length of mRNAs (1162 nt) (Fig. S7). In addition, both lncRNAs and circRNAs contained fewer exons than mRNAs (Fig. S8). Expression analysis revealed that ncRNAs exhibited lower expression levels than mRNAs in tomato flower buds (Fig. 2B).

Among all classes of ncRNAs, miRNAs are the most extensively studied. To examine their role in tomato flower buds, we predicted the target RNAs of miRNAs and assessed the correlations between their expression levels. In total, 3468 miRNA-target pairs involving mRNAs, lncRNAs, and circRNAs were identified. Of these, 551 pairs (101 miRNA-mRNA, 416 miRNA-lncRNA, and 34 miRNA-circRNA) exhibited positive correlations and 472 pairs (93 miRNA-mRNA, 350 miRNA-lncRNA, and 29 miRNA-circRNA) exhibited negative correlations, based on an absolute Pearson’s correlation coefficient of ≥0.6 and *P*-value of ≤0.05 (Fig. 2C). Competing endogenous RNA (ceRNA) activity represents a key mechanism for elucidating the regulatory interactions between ncRNAs and mRNAs. Following interaction prediction and correlation filtering, we identified 101 mRNAs, 306 lncRNAs, and 31 circRNAs as potential targets of 42 miRNAs (Fig. S9). These results suggest that the complex ncRNA-mediated regulatory networks contribute to thermotolerance in tomato and offer promising avenues for improving stress resilience through breeding programs. Nonetheless, the functional roles of the proposed ceRNA interactions under HS remain to be experimentally validated.

### Differential expression analysis of ncRNAs and mRNAs under HS

We conducted differential expression analysis of ncRNAs and mRNAs (DERs) using time-series RNA-seq data for tomato flower buds. The analysis was performed using the DESeq2 package [38], based on the 46 932 RNAs expressed in at least one sample. A total of 13 875 differentially expressed RNAs (DERs) were identified, including 8051 mRNAs, 5738 lncRNAs, 62 circRNAs, and 24 miRNAs. The largest number of DERs was observed at 0.5 h under HS, (Fig. 2D; Table S3), indicating that the most pronounced transcriptional response occurred during this early time point. The number of DERs declined at 1 h and stabilized at 3 and 5 h, suggesting that both ncRNAs and mRNAs exhibit stable expressions under prolonged exposure to HS.

To investigate the potential functions of the DERs, we categorized them into three temporal groups based on their expression patterns. A total of 8508 transcripts were classified as early DERs, showing changes at 0.5 and 1 h. Anther 2500 were categorized as late DERs, with changes at 3 and 5 h. The remaining 2867 transcripts were designated as dual DERs, characterized by sustained low expression under HS (Fig. 2E; Dataset S1). These three groups were subdivided into six clusters based on expression profiles, three early clusters (ECs), two late clusters (LCs), and one dual clusters (DC) (Fig. 2E). Among these, EC2, EC3, LC1 and LC2 exhibited activation, whereas EC1 and DC were repressed in response to HS. Gene ontology (GO) enrichment analysis was performed for each cluster (Fig. 2F; Dataset S2). EC2 showed rapid activation under HS and was significantly enriched in genes involved in the ‘response to heat’. The LCs were enriched in functions related to ‘RNA modification’ and ‘protein folding’, suggesting a role for post-transcriptional regulation and protein processing in the HS response. Finally, DC was significantly enriched in terms such as ‘nuclear division’, ‘cell cycle’, and ‘nuclear chromosome segregation’, indicating that HS conditions disrupt meiosis in tomato pollens.

### Time-specific RNA sets expressed in tomato flower buds under HS

Time-specific expression of mRNAs and ncRNAs plays a critical role in regulating plant responses to HS. To transcripts uniquely expressed at specific time points, we analyzed 30 247 RNAs, including 21 236 mRNAs, 5946 lncRNAs, 2693 circRNAs, and 372 miRNAs, with the expression values >1 at least one time point. A time specificity (TS) scoring algorithm (see Materials and methods) was applied, and transcripts with a TS score >0.3 were considered uniquely expressed at a given time point. Based on this threshold, we identified 6849 time-specific RNAs across the five time points, comprising 3411 mRNAs, 2272 lncRNAs, 1087 circRNAs, and 79 miRNAs. Further analysis revealed substantial variations in the number of time-specific transcripts across time points (Fig. S10). The highest number was observed at 0 h (2410, accounting for 35.19% the total). while the lowest was detected at 3 h under HS (334, 4.89%). The variable number and proportion of time-specific RNAs indicated that the regulation of mRNAs and ncRNAs expressions contributes to increase tomato heat tolerance in a time-oriented manner.

### Coexpression network analysis among time-series stages of HS

**Figure 3:**
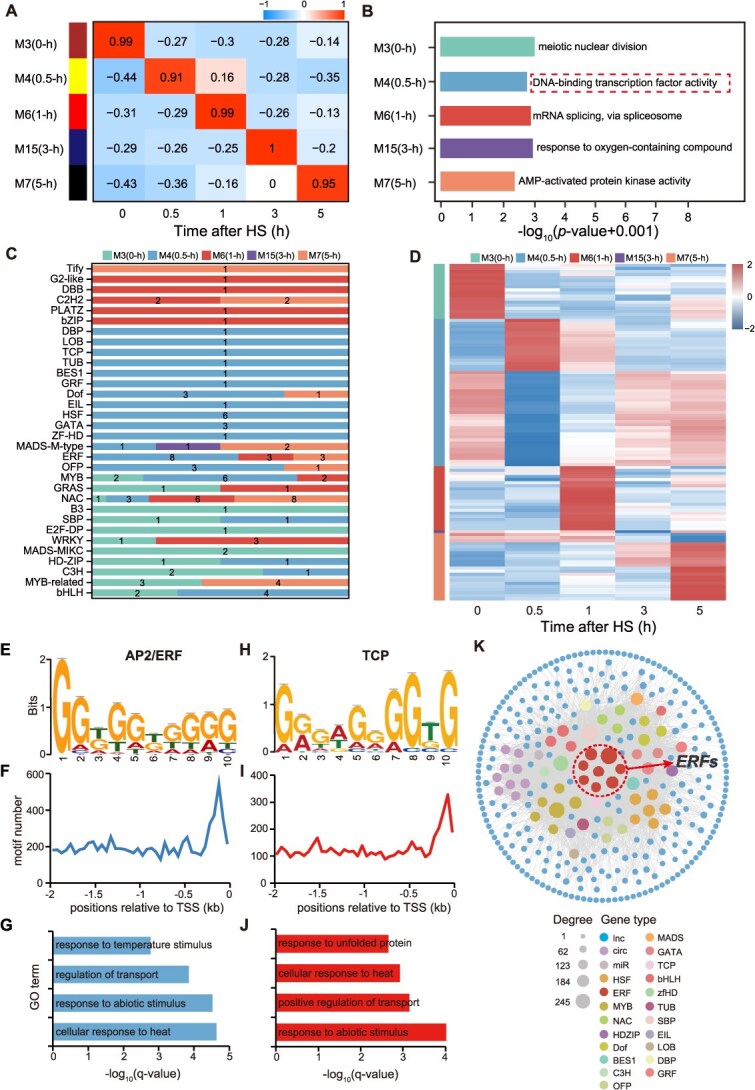
Coexpression modules identified using WGCNA. (A) Heatmap showing the correlation coefficients and *P*-value of the time-specific modules. M3, module 3. M4, module 4. M6, module 6. M15, module 15. M7, module 7. (B) Most significant enriched functional terms associated with each time-specific module. (C) Distribution of TF families and their member counts across the time-specific modules. Numbers indicate the number of TF members within each family in the corresponding module. (D) Heatmap of scaled FPKM values for the 110 TFs. (E, H) Motif1 and motif2 enriched among the M4 genes. (F, I) Position frequencies of the same motifs determined as number of motifs per 50-bp bins in the promoters. (G, J) GO enrichment of the candidate target genes. (K) Co-expression network based on correlation among TFs in the module that correlated with 0.5-h period.

The regulatory relationships between mRNAs and ncRNAs across different stages of HS can be explored through coexpression network analysis. To investigate potential regulatory networks associated with tomato thermotolerance, we analyzed 30 247 expressed RNAs by using weighted gene coexpression network analysis (WGCNA) [39]. RNAs with low coefficient variation (<0.5) across all five time points were excluded. After filtering, 9618 RNAs, including 4399 mRNAs, 2602 lncRNAs, 2513 circRNAs, and 104 miRNAs met the inclusion criteria. This analysis identified 19 coexpression modules (Fig. 3A; Fig. S11A), each containing between 44 (M19 module) and 2535 (M1 module) RNAs (Fig. S11B). We examined the correlation between each module and the five time points based on expression patterns of both mRNAs and ncRNAs. Among the 19 modules, 5 showed strong correlations (*r*^2^ > 0.9) with specific time points (Fig. 3A), suggesting that the RNAs within these modules are functionally associated with distinct stages of the HS response. To further investigate this relationship, we compared the five time-specific modules with the previously identified time-specific expression sets. Between 51.45% and 85.80% of the RNAs in each module overlapped with the corresponding time-specific sets (Table S4), indicating that these modules captured a substantial portion of the temporally regulated transcripts. Taken together, these results suggested that the mRNAs and ncRNAs in these coexpression modules play essential roles in regulating the HS response in tomato flower buds.

**Figure 4:**
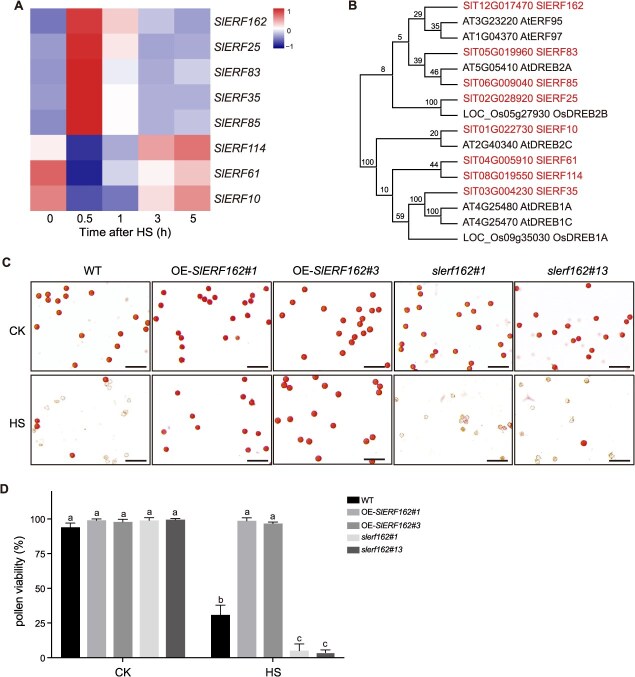
*SlERF162-*mediated modulation of basal thermotolerance. (A) Heatmap showing the expression levels of eight ERF*s* in the 0.5-h-correlated module. (B) Phylogenetic analysis of ERF proteins from *Arabidopsis thaliana* (At), tomato (*S. lycopersicum*, Sl), and rice (*Oryza sativa*, Os). (C) Acetocarmine staining of mature pollen grains in WT, OE-*SlERF162*, and *slerf162* lines under CK and HS. Bars = 100 μm. (D) Pollen viability in WT, OE-*SlERF162*, and *slerf162* lines under CK and HS. Values represent the mean ± SD (*n* = 5). Different letters indicate statistically significant differences at *P* < 0.05 (two-way ANOVA and Tukey’s multiple comparisons test). CK, control check. HS, heat stress. OE, overexpressing. WT, wild type.

### Identification of HS-related TFs in tomato flower buds

To examine the biological processes associated with tomato thermotolerance within the coexpression modules, we conducted functional classification of mRNAs from all coexpression modules by using TBtools software [40] (Fig. 3B; Fig. S12; Dataset S3). Notably, the module correlated with the 0-h time point (M3) was significantly enriched in genes involved in ‘meiotic nuclear division’, consistent with the developmental stage of 4-mm tomato flower buds (Fig. 3B). In contrast, the module correlated with 0.5 h (M4) was enriched in ‘DNA-binding transcription factor activity’, suggesting that TFs may play key roles in the early HS response. To further investigate the contribution of TFs in these time-specific modules, we identified 110 putative TFs spanning 32 families, including the HSF, ERF, bZIP, and MYB (Fig. 3C; Table S5). Expression profiling revealed that 75 of these TFs exhibited high expression during specific time periods, whereas 35 showed low expression levels (Fig. 3D). To understand the regulatory mechanism involved in the early-stage response to HS, we focused on *cis*-regulatory elements in the 0.5-h-correlated module. Using MEME software [41], we identified overrepresented 10-bp motifs within the −2-kb promoter regions of the associated genes. These motifs were analyzed using TOMTOM [42] and retained only if they matched known JASPAR CORE plant motifs with a *q*-value <0.05. Two enriched motifs were identified: one matching the binding site of ERFs and another corresponding to TCP family. The ERF motif was present in the promoters of 456 genes, whereas the TCP motif was detected in the promoters of 266 genes (Fig. 3E and H). These motifs were preferentially located upstream of the transcription start site (Fig. 3F and I). GO enrichment analysis revealed that the corresponding genes were significantly associated with responses to temperature stimulus, regulation of transport, response to abiotic stimulus, and cellular response to heat (Fig. 3G and J). According to the coexpression network analysis, ERF genes occupied central positions within the regulatory network (Fig. 3K). Based on these findings, we selected ERFs for further investigation.

**Figure 5:**
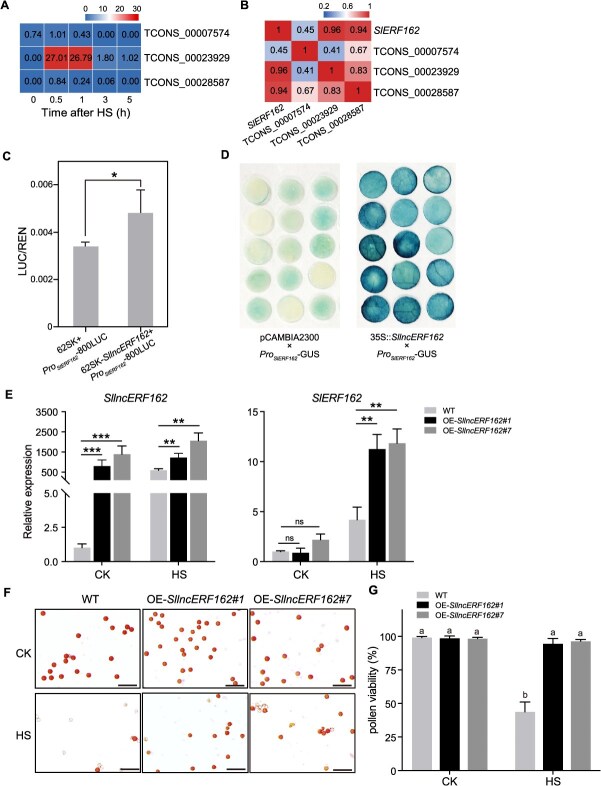
*SllncERF162* functions upstream of *SlERF162* to enhance basal thermotolerance. (A) Heatmap showing the expression levels of three lncRNAs across different time points. Number within each cell represent FPKM values. (B) Correlation coefficients between *SlERF162* and the three lncRNAs. (C) Dual-luciferase reporter (DLR) assays demonstrating that *SllncERF162* binds to the promoter of *SlERF162* and activates its expression. Values represent the mean ± SD (*n* = 3). Student’s *t*-test, ^*^P < 0.05. (D) GUS staining assay confirming the interaction between *SllncERF162* and the *SlERF162* promoter. (E) Expression levels of *SllncERF162* and *SlERF162* in the OE-*SllncERF162* plants under CK and HS. Values represent the mean ± SD (*n* = 3). Asterisks indicate the significant differences (^**^P < 0.01, ^***^P < 0.001), ns, not significant (two-way ANOVA and Tukey’s multiple comparisons test). (F) Acetocarmine staining of mature pollen grains in the WT and OE-*SllncERF162* plants under CK and HS. Bars = 100 μm. (G) Pollen viability in the WT and OE-*SllncERF162* plants under CK and HS. Values represent the mean ± SD (*n* = 5). Different letters indicate statistically significant differences at *P* < 0.05 (two-way ANOVA and Tukey’s multiple comparisons test). CK, control check. HS, heat stress. OE, overexpressing. WT, wild type.

**Figure 6:**
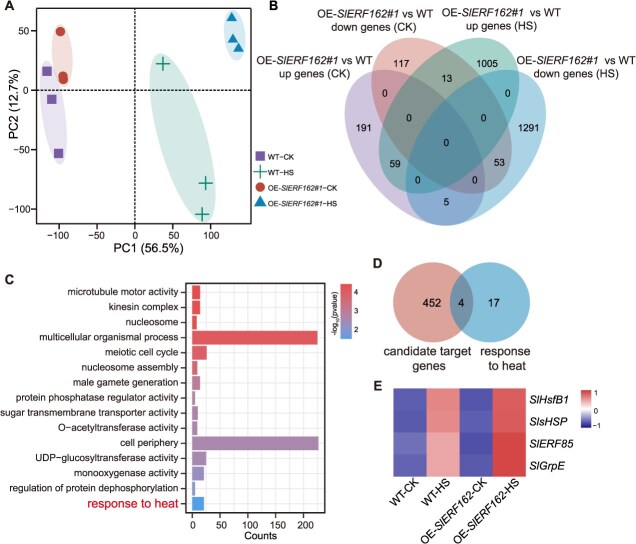
Transcriptomic analysis of *SlERF162* regulated genes in tomato flower buds under HS. (A) PCA of OE-*SlERF162#1* and WT plants under CK and HS. (B) GO enrichment analysis of DEGs in OE-*SlERF162#1* plants under HS. (C) Venn diagram showing the overlap and distribution of DEGs in response to HS between the OE-*SlERF162#1* and WT plants. (D) Identification of candidate genes potentially regulated by *SlERF162* under HS. (E) Heatmap displaying the expression levels of five candidate genes regulated by *SlERF162*. CK, control check. HS, heat stress. OE, overexpressing. WT, wild type.

### *SlERF162* enhances the tomato basal thermotolerance during pollen development

Within the 0.5-h-correlated module, eight ERFs were predicted to bind target gene promoters. Among them, *SlERF25* (*SlT02G028920*), *SlERF35* (*SlT03G004230*), *SlERF83* (*SlT05G019960*), *SlERF85* (*SlT06G009040*), and *SlERF162* (*SlT12G017470*) were significantly upregulated at 0.5 h under HS, whereas *SlERF10* (SlT01G022730), *SlERF61* (*SlT04G005910*), and *SlERF*114 (*SlT08G019550*) were markedly downregulated (Fig. 4A; Fig. S13). Phylogenetic analysis revealed that *SlERF162* shares high sequence homology with *AtERF95* and *AtERF97*, both of which have been implicated in enhancing basal thermotolerance in *Arabidopsis* [18]. We conducted RT-qPCR analysis to examine the expression levels of *SlERF162* in leaf, stem, root, 4-mm flower buds, 6-mm flower buds, 8-mm flower buds, fully open flowers, mature green fruits, and red ripe fruits. The results showed that *SlERF162* is not a tissue-specific expressed gene, as it was detected in all examined tissues with comparable expression levels (Fig. S14A). Subcellular localization assays showed that *SlERF162* is localized in the nucleus (Fig. S14B). To further validate the role of *SlERF162* in tomato, we generated overexpression plants (OE-*SlERF162*) and knockout mutants (*slerf162*) (Fig. S15). Acetocarmine staining showed that both transgenic lines maintained normal pollen viability under control conditions (CK) (Fig. 4C). However, under HS, OE-*SlERF162* plants exhibited significantly higher pollen viability (98.6%) than the wild type (WT) (30.8%). In contrast, the knockout *slerf162* lines displayed extensive pollen adhesions and collapsed, with viability reduced to 5.1% and 3.7% respectively (Fig. 4C and D). These results suggested that *SlERF162* plays a protective role in maintaining pollen viability under HS.

### *SllncERF162* acts at the upstream of *SlERF162* to enhance basal thermotolerance of tomato flower buds

LncRNAs play essential roles in regulating gene expression in plant responses to biotic and abiotic stresses [43]. In this study, we identified 5738 differentially expressed lncRNAs (Fig. 2D), among which 22 showed strong sequence complementarity with *SlERF162* (Fig. S16; Table S6). Based on this observation, we hypothesized that lncRNAs potentially regulate the expression level of *SlERF162*. Pairwise analysis identified three candidates, including *TCONS_00007574*, *TCONS_00023929*, and *TCONS_00028587*, as potential regulators of *SlERF162* in the early-stage cluster EC2. Among these, *TCONS_00023929* exhibited a higher expression level under HS (Fig. 5A; Fig. S17), and the strongest positive correlation with *SlERF162* expression (Fig. 5B). The RT-qPCR results confirmed that *TCONS_00023929* was significantly upregulated by HS, with the expression peaking at 0.5 h (Fig. S18). These findings suggested that *TCONS_00023929* may act as a positive regulator of *SlERF162* under HS. Therefore, we designated this transcript as *SllncERF162*.

To investigate the regulatory relationship between *SlERF162* and *SllncERF162*, we conducted transient dual-luciferase reporter (DLR) assay and β-glucuronidase (GUS) staining assay. The results demonstrated that *SllncERF162* can directly bind to the promoter region of *SlERF162* (Fig. 5C and D). To further examine the function of *SllncERF162*, we generated overexpression plants. Under HS, *SlERF162* expression was significantly elevated in the *OE-SllncERF162* plants compared with WT plants; however, no difference in expression was observed under CK (Fig. 5E). Microscopic analysis revealed that pollen viability was markedly higher in the OE*-SllncERF162* plants than in the WT under HS (Fig. 5F and G). These findings suggested that *SllncERF162* enhances basal thermotolerance in tomato by directly regulating the expression of *SlERF162* during pollen development.

### *SlERF162* modulates basal thermotolerance through HS-responsive genes

To examine the HS-responsive pathways regulated by *SlERF162*, we conducted transcriptome profiling of WT and OE-*SlERF162* plants after 0.5 h of HS. The PCA results revealed clear separation between the CK and HS samples (Fig. 6A). Comparative expression analysis identified 1005 upregulated and 1291 downregulated genes in the OE-*SlERF162* plants, whereas gene expression in the WT showed less sensitivity to HS (Fig. 6B; Dataset S4). GO enrichment analysis revealed that these differentially expressed genes (DEGs) were significantly associated with pathways such as ‘response to heat’, ‘meiotic cell cycl,e’, and ‘UDP-glucosyltransferase’ (Fig. 6C, Dataset S5). These results suggest that the DEGs are likely regulated by *SlERF162* and contribute to the HS response in tomato.

**Figure 7:**
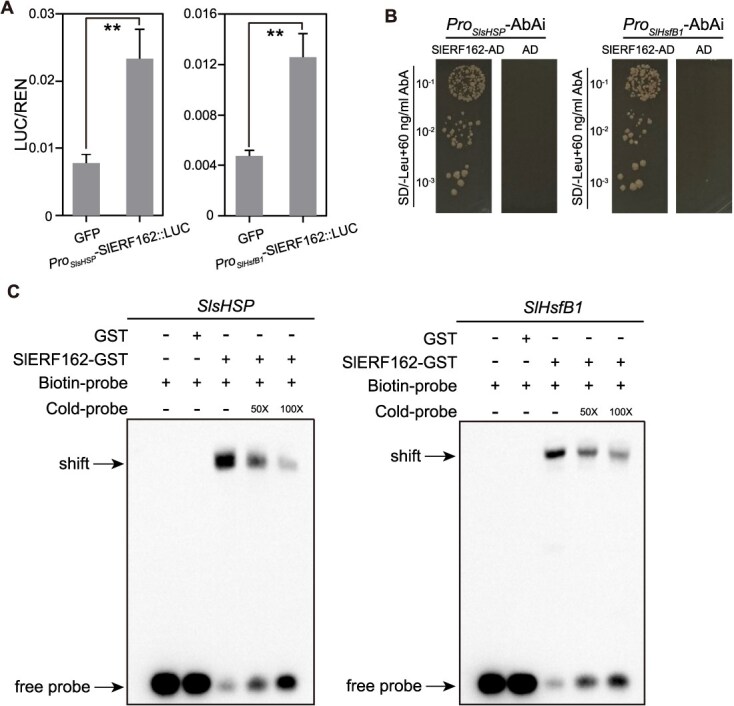
SlERF162 binds to the promoters of *SlsHSP* and *SlHsfB1*. (A) DLR assays showing that SlERF162 binds to the promoters of *SlsHSP* and *SlHsfB1*, activating their transcription. Values represent the mean ± SD (*n* = 3). Student’s *t*-test, ^**^P < 0.01. (B) Y1H analysis confirming the interaction between *SlERF162* and the promoters of *SlsHSP* and *SlHsfB1*. (C) EMSA demonstrating the direct binding of SlERF162 to the promoter regions of *SlsHSP* and *SlHsfB1*. GST, glutathione-S-transferase.

We next examined 21 genes annotated under the ‘response to HS’ category and identified four potential targets of *SlERF162* based on the predicted TF-binding interactions (Fig. 6D; Dataset S6). These four genes included an HSF, an HSP, an ERF, and a *GrpE* (*GroP-like gene E*). All four were strongly induced in the OE-*SlERF162* plants under HS (Fig. 6E). To validate these regulatory relationships, we performed DLR assays, yeast one-hybrid (Y1H), and electrophoretic mobility shift assays (EMSA). The results confirmed that *SlERF162* directly binds to the promoters of *SlHsfB1* and *SlsHSP* (Fig. 7). The RT-qPCR results further showed that the expression levels of *SlHsfB1* and *SlsHSP* were significantly higher in the OE-*SlERF162* plants and reduced in the *slerf162* lines compared with the WT under HS (Fig. S19). Together, these results indicated that *SlERF162* directly activates *SlHsfB1* and *SlsHSP* transcription in response to HS in tomato.

## Discussion

### Dynamic whole-transcriptome analysis identifies temporal modules of HS response in tomato

To date, no study has reported closely spaced time-series transcriptomic data to investigate the HS response in tomato. In this study, we generated whole-transcriptome data from 15 samples collected across five time points, providing a detailed temporal resolution gene expression dynamics. A total of 13 875 DERs were identified in response to HS and classified into three temporal categories, ES, LS, and DS (Fig. 2E). Functional enrichment analysis revealed distinct biological processes associated with each stage, including ‘response to heat’ in the ES, ‘protein folding’ in the LS, and ‘nuclear division’ in the DS (Fig. 2F). The regulatory networks containing mRNA, lncRNA, circRNA, and miRNA under HS have been revealed in maize [44] and cucumber [45]. In this study, we identified the mRNA, lncRNA, circRNA, and miRNA that responded to HS on a genome-wide scale in tomatoes. This time-series transcriptomic atlas offers valuable insight into the regulatory mechanisms underlying basal thermotolerance in tomato.

### *SllncERF162-SlERF162* is a novel transcriptional module involved in tomato basal thermotolerance

**Figure 8:**
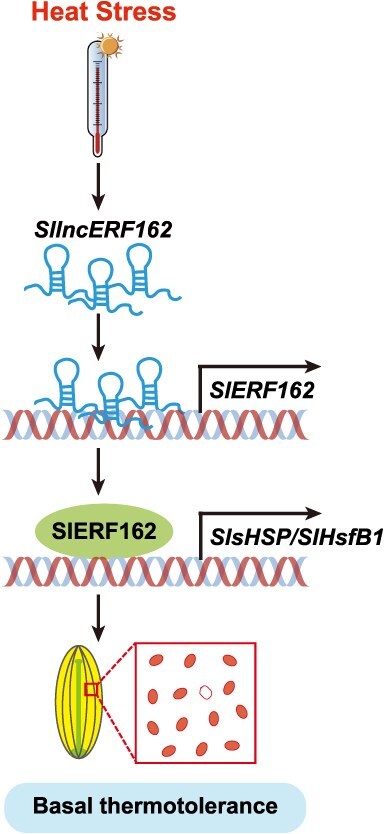
Proposed model of the *SllncERF162-SlERF162* regulatory module in tomato basal thermotolerance.

In the contexts of global warming and climate change, understanding plant responses to HS and developing thermotolerant crop varieties have become top priorities for sustainable agriculture. To mitigate the effects of HS, plants have evolved complex defense mechanisms, which included stabilization of cellular membrane, enhanced reactive oxygen species (ROS) scavenging, and activation of calcium signaling and molecular chaperone response systems [46]. However, the molecular basis of HS response in tomato remains incompletely understood. The present study revealed the comprehensive genome-wide expression profile of total RNAs and constructed coexpression networks in tomato flower buds under HS. Our findings support the existence of a novel ncRNA regulatory module, comprising *SllncERF162* and *SlERF162*, that plays a critical role in regulating basal thermotolerance during pollen development in tomato (Fig. 8).

### *SlERF162* enhances thermotolerance in tomato and may influence plant growth and development

ERFs are well-established regulators of plant responses to abiotic stress and are believed to contribute to thermotolerance by modulating the activity of HSFs. For instance, brassinosteroid-mediated *ERF49* enhances heat tolerance in *Arabidopsis* by regulating the expression of *HSFA2* and *HSPs* [47]. Similarly, *BdERF014* coevolves with a cluster of small HSPs and directly regulates this cluster in response to HS [48]. In *Lilium*, the heat-induced *ERF012*, a member of the DREB-A5 subfamily, promotes thermotolerance by directly regulating *LlHsfB1*, *LlHsfA1*, *LlHsfA2*, *LlHsfA3a*, and *LlHsfA3b* [49]. In the present study, we identified *SlERF162*, a heat-induced homolog of *AtERF95* and *AtERF97*, as a positive regulator of tomato basal thermotolerance. *SlERF162* directly activates the expression of *SlHsfB1* and *SlsHSP*, enhancing the plant’s HS response (Fig. 6E and F; Figs S14, S18). In contrast, the expression of *AtERF95* and *AtERF97* is barely regulated by HS in *Arabidopsis* [18]. Although *AtERF95* and *AtERF97* function as dimers to regulate downstream targets and confer basal thermotolerance, whether *SlERF162* interacts with other ERFs or additional regulatory genes in tomato remains to be determined. These findings highlight possible species-specific regulatory mechanisms and suggest that members of the ERF family may be regulated at both transcriptional and post-transcriptional levels in response to HS. In addition, we also observed SlERF162’s effects on plant development. During the vegetative growth stage, OE-*SlERF162* plants exhibited significantly reduced height compared to WT (Fig. S20A), accompanied by pronounced alterations in leaf morphology. The OE-*SlERF162* plants displayed markedly smaller leaves with an elongated shape, pronounced downward curling, and substantially reduced leaf lobing compared to WT (Fig. S20B). Intriguingly, such developmental alterations were not observed in *SlERF162* knockout lines. During the reproductive growth phase, OE-*SlERF162* plants showed no significant floral abnormalities relative to WT. In contrast, *SlERF162* knockout lines exhibited mild stamen malformations (Fig. S20C). These phenotypic changes suggest *SlERF162* may participate in growth–defense trade-offs. Future studies should investigate whether *SlERF162* influences plant growth and development roles, particularly its interaction with phytohormone signaling networks.

### *SllncERF162* positively regulates SlERF162 expression level to enhance thermotolerance in tomato

LncRNAs play essential roles in plant responses to various abiotic stresses, including heat, cold, and salinity [50]. For example, the intergenic lncRNA *MtCIR2* enhances freezing tolerance by modulating the *CBF/DREB1* gene cluster in *Medicago truncatula* [51]. In potato, the antisense lncRNA *StFLORE* improves drought tolerance by reducing water loss [52]. In *Populus trichocarpa*, the salt-induced *Ptlinc-NAC72* recognizes tandem GAAAAA motifs in the 5′-UTR of *PtNAC72.A*/*B*, directly promoting their expression [53]. Similarly, in *Betula platyphylla*, the salt-responsive lncRNA *BplncSIR1* binds to the promoter of *BpNAC2* and enhances its expression, contributing to salt tolerance [54]. In cotton, the salt-induced lncRNA *TRABA* represses the activity of the *GhBGLU24* promoter under salt stress, which encodes a β-glucosidase [55]. In this study, we identified a heat-induced lncRNA, *SllncERF162*, which acts as a positive regulator of *SlERF162* expression. These two transcripts are located on different chromosomes, *SlERF162* on chromosome 12 and *SllncERF162* on chromosome 3, indicating a potential transregulatory relationship. Previous studies have shown that lncRNAs can regulate gene expression through RNA–DNA triplex formation [56, 57]. For instance, in *Arabidopsis*, the lncRNA *APOLO* forms R-loops and interacts with the TF *WRKY42* to modulate its binding to the *RHD6* promoter, a key regulator of root hair development under cold stress [58]. Based on these findings and our results, we propose that *SllncERF162* may recognize the promoter of *SlERF162* through R-loop formation, thereby enhancing its transcription under HS. However, this proposed mechanism requires further experimental validation. Collectively, our findings offer new insights into the regulatory roles of lncRNAs in response to abiotic stress in tomato.

In summary, we constructed a comprehensive RNA expression atlas of tomato flower buds under HS, offering a valuable resource for the discovery of stress-responsive genes and ncRNAs. Our findings highlight the critical role of the *SllncERF162–SlERF162* regulatory module in promoting thermotolerance during pollen development. The candidate genes and ncRNAs identified in this study can serve as valuable targets for developing heat-tolerant tomato cultivars.

## Materials and methods

### Plant materials and growth conditions

The experiment utilized *S. lycopersicum* accession Heinz 1706. Seeds were cultivated in nursery substrate-filled pots for ~4 weeks. The five-leaf expanded tomato seedlings were transplanted into a 1-l plastic pot. The growth conditions were controlled at 25/20°C (day/night) with a photoperiod of 16 h, 600 μmol m^−2^ s^−1^ photosynthetic photon flux density (PPFD) and 8-h dark, and relative humidity of 55%–65%. After transplantation, tomato plants were irrigated with 1 g/l Huawuque water-soluble fertilizer every 3 days. Flower bud samples were collected at the two-to-three inflorescence stage (0 h) as three biological replicates. The remaining plants were exposed to 37°C for 0.5, 1, 3, and 5 h. Flower buds from both control and HS groups were snap-frozen in liquid nitrogen and stored at −80°C for subsequent RNA extraction.

### Strand-specific library construction and sequencing

Total RNA was isolated using the Quick RNA Isolation Kit (Huayueyang Biotechnology) and quantified using a Nanodrop 2000 spectrophotometer (Thermo Fisher Scientific, Delaware, USA). rRNA-depleted RNAs (mRNA/ncRNA) were fragmented and reverse-transcribed using random primers. Second-strand cDNA was synthesized with DNA polymerase I, RNase H, dNTP (dUTP instead of dTTP), and the appropriate buffer, followed by purification (QiaQuick PCR kit). After end repair, poly(A) addition, and Illumina adapter ligation, second-strand cDNA was digested with UNG (Uracil-N-Glycosylase). Size-selected fragments were PCR-amplified and sequenced on Illumina NovaSeq6000. Raw reads were filtered to remove adapters and low-quality sequences prior to analysis.

### Small RNA libraries construction and sequencing

RNA samples meeting quality criteria (OD 260/280: 1.8–2.2; RIN ≥ 7.5) were used for library preparation with the Small RNA Sample Pre Kit. Following adapter ligation and cDNA synthesis, target fragments (140–160 bp, representing 18–30 nt small RNAs plus adapters) were size-selected by 8% PAGE. Libraries were sequenced on an Illumina HiSeq2000, generating 50-bp single-end reads.

### Identification of mRNA, lncRNA, and circle RNA

Clean reads from strand-specific libraries were aligned to the tomato genome sequence (SLT1.0) using TopHat version 2.1.1, with known mRNAs identified based on genome annotations. Novel transcripts were evaluated for protein-coding potential using CNCI [59], CPC (http://cpc.cbi.pku.edu.cn,), and Pfam. Transcripts identified by all three methods were classified as lncRNAs. Their expression levels were quantified using Cuffdiff, with results expressed in FPKM. Differentially expressed lncRNAs and mRNAs were identified by DESeq2. 20-mers from both ends of the unmapped reads were aligned to the reference genome to locate splice-site anchors. Reverse-oriented anchor pairs (head-to-tail) were analyzed with find_circ [36] to identify circRNAs. Validated breakpoints required flanking GU/AG splice motifs following read alignment extension. CircRNA levels were measured using back-spliced junction reads normalized to RPM (reads per million mapped reads).

### Analysis of small RNA-seq data

Adapter trimming (cutadapt 4.1) was followed by quality filtering to remove reads with ambiguous bases, poly A sequences, or lengths outside 18–30 nt. The Rfam database (release 14.5) [60] were used to annotate and exclude rRNA, tRNA, snRNA, and sRNA sequences. An investigation of the read length distribution was performed to confirm that the majority of the remaining reads were mature miRNA sequences. After confirming miRNA-like size distribution, high-quality reads were aligned to the tomato reference genome (SLT1.0) using Bowtie software [61]. Novel miRNAs were predicted using MiRDeep2 [62] with significant randfold *P*-values <0.05 and miRDeep2 scores >4. The expression levels of the identified known and potentially novel miRNAs were normalized into RPM (read counts per million mapped reads).

### Identification of time-specific gene sets

Time-specific genes were identified using a TS (time-specific) scoring algorithm [63, 64], which compares a gene’s expression at each time point against its maximum expression across all other time points. For a given gene *i* with expression values *EV_i_* = (*Ei 1*, *Ei 2*, *Ei 3*, . . ., *Ei 5*), and the TS score at time point *j* is calculated as: TS (*i*, *j*) = $1-\frac{\max\{\mathrm{E}}_k^i}{{\mathrm{E}}_j^i}$, where$1\le k\le 5$, *k*  $\ne$  *j*. Scores range from 0 to 1, with higher values indicating greater time-specific expression.

### Gene network construction and visualization

Scale-free coexpression network analysis was performed on log2 transformed FPKM/RPM values of expressed RNAs using the WGCNA package (v 1.70) in R [39]. RNAs with low FPKM/RPM (FPKM/RPM < 1 in all samples or only expressed in one sample) or low coefficient of variation of FPKM/RPM (CV < 0.5 among five time points) were filtered out. Networks were built using a soft threshold power of 9 and a dynamic treecut algorithm with a minimum cluster size of 30 and merging threshold of 0.25. RNAs were clustered into 19 modules. The networks were visualized using Cytoscape v.3.9.0.

### Identification of *cis*-motifs

*De novo* motif discovery in the promoter regions (2 kb upstream of transcription start sites) of yellow module genes was performed using MEME. Genomic sequences were extracted using a custom Python script. From the 10 motifs initially identified (Motifs 1–10). those with an *E*-value >10^−5^ were discarded. The remaining motifs were compared against the JASPAR CORE Plantae database [65] using TOMTOM, retaining only matches with significant similarity (*q*-value < 0.05).

### RNA extraction and RT-qPCR analysis

Single-stranded cDNA was prepared using the PrimeScript RT Reagent Kit with gDNA Eraser (TaKaRa). RT-qPCR was performed in a 10-μl reaction volume consisting of 5 μl TB Green Premix Ex Taq (Tli RNaseH Plus) (TaKaRa), 0.25 μl ROX Reference Dye (50×), 0.25 μl each of forward and reverse primers (10 μM), 1 μl cDNA templates, and 3.25 μl purified water. Amplification was performed on a QuantStudio Flex 6 system (Applied Biosystems) with the following protocol: 95°C for 30 s, followed by 40 cycles of 95°C for 5 s and 60°C for 34 s. All reactions were run in triplicate, with relative expression calculated by the 2^−ΔΔCt^ method. Expression levels for all assayed genes were normalized using *SlCAC* (*Solyc08g006960*). All the primers are listed in Dataset S7.

### Subcellular localization

The CDS sequence of SlERF162 lacking the stop codon was cloned into the pCAMBIA-super1300 vector fused with green fluorescent protein (GFP). For transient expression, the recombinant plasmid was transformed into *Agrobacterium tumefaciens* strain GV3101, and the bacterial cells were resuspended in an infiltration buffer containing 0.1 mM Acetosyringone, 10 mM MgCl₂, and 10 mM MES. The suspension was infiltrated into *Nicotiana benthamiana* leaves, with the empty pCAMBIA-super1300 vector used as a positive control. Subcellular localization of the SlERF162-GFP fusion protein in leaf epidermal cells was observed using a ZEISS LSM900 confocal microscope 48 h post-infiltration.

### Overexpression and mutant plants construction

To generate the *SlERF62* overexpressing vector, the full-length cDNA sequence for *SlERF62* was amplified and ligated into the pFGC1008-HA vector. To generate the *SllncERF162* overexpressing vector, the full-length cDNA sequence of *SllncERF162* was cloned into the pRI101 vector. Independent guide RNA sequences of *SlERF62* were designed using the web application tool CRISPR-P (http://cbi.hzau.edu.cn/cgi-bin/CRISPR2/SCORE) and cloned into the pCP041 vector to construct the CRISPR/Cas9 genome editing plasmid vector of *SlERF62*. The destination vectors were transformed into the Ailsa Craig via *Agrobacterium*-mediated transformation.

### Y1H assays

The full-length CDS of *SlERF162* was cloned into the pGADT7 and 2-kb promoter sequences of target gene were cloned into the pAbAi vectors. They were cotransformed into yeast cells. Whereafter Y1HGold yeast cells containing the fusion vectors pAbAi were coated on synthetic dropout medium lacking Ura (SD/-Ura) with different Aureobasidin A (AbA) concentrations, and the optimum concentration was found in the medium where yeast cells are no longer growing. The fusion vectors pGADT7 were transferred into the above yeast cells. When yeast cells are grown in a synthetic dropout medium lacking Leu (SD/−Leu) with the optimum concentration of AbA, transcription factors interacted with promoters.

### Dual-luciferase reporter assays

The full-length cDNA sequence of *SlERF162* or *SllncERF162* were cloned into the pGreenII 62-SK vectors as effectors, and the 2-kb promoter sequence of target genes were cloned into pGreenII800LUC vectors using a Seamless Assembly Cloning Kit (Clone Smarter) as reporters. The fusion vectors were transformed into *Agrobacterium* strain GV3101 via heat-shock. Bacterial cultures were resuspended in infiltration buffer (0.1 mM acetosyringone, 10 mM MgCl_2_, and 10 mM MES) to identical concentrations of 0.8–1.2 (OD600). Equal volumes of bacterial suspensions were coinfiltrated into *N. benthamiana* leaves. Two days after infiltration, leaves were collected for the dual-LUC assay using the Dual Luciferase Reporter Assay Kit (Vazyme). Firefly LUC and Renilla (REN) activities were measured using SpectraMax i3x instrument, with results expressed as LUC/REN ratios. More than three biological repeats were measured for each the experiment group.

### Electrophoretic mobility shift assay

The biotin-labeled DNA probes containing the putative binding sequence were synthesized (Shanghai Sangon Biotech) and annealed into double chains. The GST-SlERF162 fusion protein was purified by GST magnetic beads (Shanghai Yeasen Biotechnology, 20562ES). Binding reactions were performed by LightShift EMSA Optimization and Control Kit (Thermo Scientific, 20 148X). Protein-DNA complexes were resolved on a 6% nondenaturing polyacrylamide gel in 0.5× TBE buffer at 100 V for 1 h at 4°C. Subsequently, DNA was transferred to a nylon membrane (Cytiva, Hybond-N+) and crosslinked by UV irradiation. Biotin-labeled probes were detected using Chemiluminescent Nucleic Acid Detection Module (Thermo Scientific, 89 880). The sequences of DNA probes used were listed in Dataset S7.

### GUS staining

The full-length cDNA sequence of *SllncERF162* was cloned into the pRI101 vector and the 2-kb promoter sequence of *SlERF162* was cloned into pCAMBIA2300 vector using a Seamless Assembly Cloning Kit (Clone Smarter). *Agrobacterium* transformation and tobacco infiltration refer to the above methods in Dual-luciferase assays. Tobacco leaves were drilled with a hole punch and soaked in GUS dye for 12 h and 75% ethanol for 3 days. More than three biological repeats were measured for each the experiment group.

### Pollen viability analysis

To investigate pollen viability *in vitro*, mature pollen grains from dehiscent anthers of the tomato flowers in full bloom were stained by Acetocarmine (Huayueyang, http://www.huayueyang.com/). At least five fields were taken for each flower. Pollen viability was measured as the ratio of normally stained pollen grains to total pollen counts. As for HS condition, tomato plants with at least one inflorescence are subjected to 37°C for 24 h. After treatment, unopened flowers are marked, and pollen is collected from these flowers upon opening for staining and observation.

## Statistical analysis

For each experiment, a minimum of three independent biological replicates (separate sampling events under identical conditions) were analyzed. Data are presented as mean ± SD, with asterisks denoting statistical significance. Analyses were performed using Student’s *t*-test, one-way ANOVA, or two-way ANOVA in GraphPad Prism 9.0.0 (GraphPad Software, San Diego, CA, USA) (Dataset S8).

## Accession numbers

The accession numbers for genes in this study are as follows: *SlHsfA1* (SlT03G019230), *SlHsfA2* (SlT08G011830), *SlHsfB1* (SlT02G028970), *SlsHSP* (SlT12G015660), *SlERF10* (SlT01G022730), *SlERF25* (SlT02G028920), *SlERF35* (SlT03G004230), *SlERF61* (SlT04G005910), *SlERF83* (SlT05G019960), *SlERF85* (SlT06G009040), *SlERF114* (SlT08G019550), *SlERF162* (SlT12G017470), *SlGrpE* (SlT12G006520), and *SllncERF162* (TCONS_00023929).

## Supplementary Material

Web_Material_uhaf205

## Data Availability

All data supporting this study are included in the manuscript and Supplementary Materials. Raw sequencing data have been deposited in the Genome Sequence Archive (GSA) of the Beijing Institute of Genomics (BIG) Data Center (https://bigd.big.ac.cn/) under accession number CRA018919.
