# Supplementary material for: Genome-wide expression atlas of tomato flower buds revealed the SllncERF162-SlERF162 module associated with basal thermotolerance
Source: Hortic Res. 2025 Jul 31;12(11):uhaf205. doi: 10.1093/hr/uhaf205 (PMC12574544; doi:10.1093/hr/uhaf205)
Supplement: Web_Material_uhaf205 [file web_material_uhaf205.zip › Supplementary figures.docx]

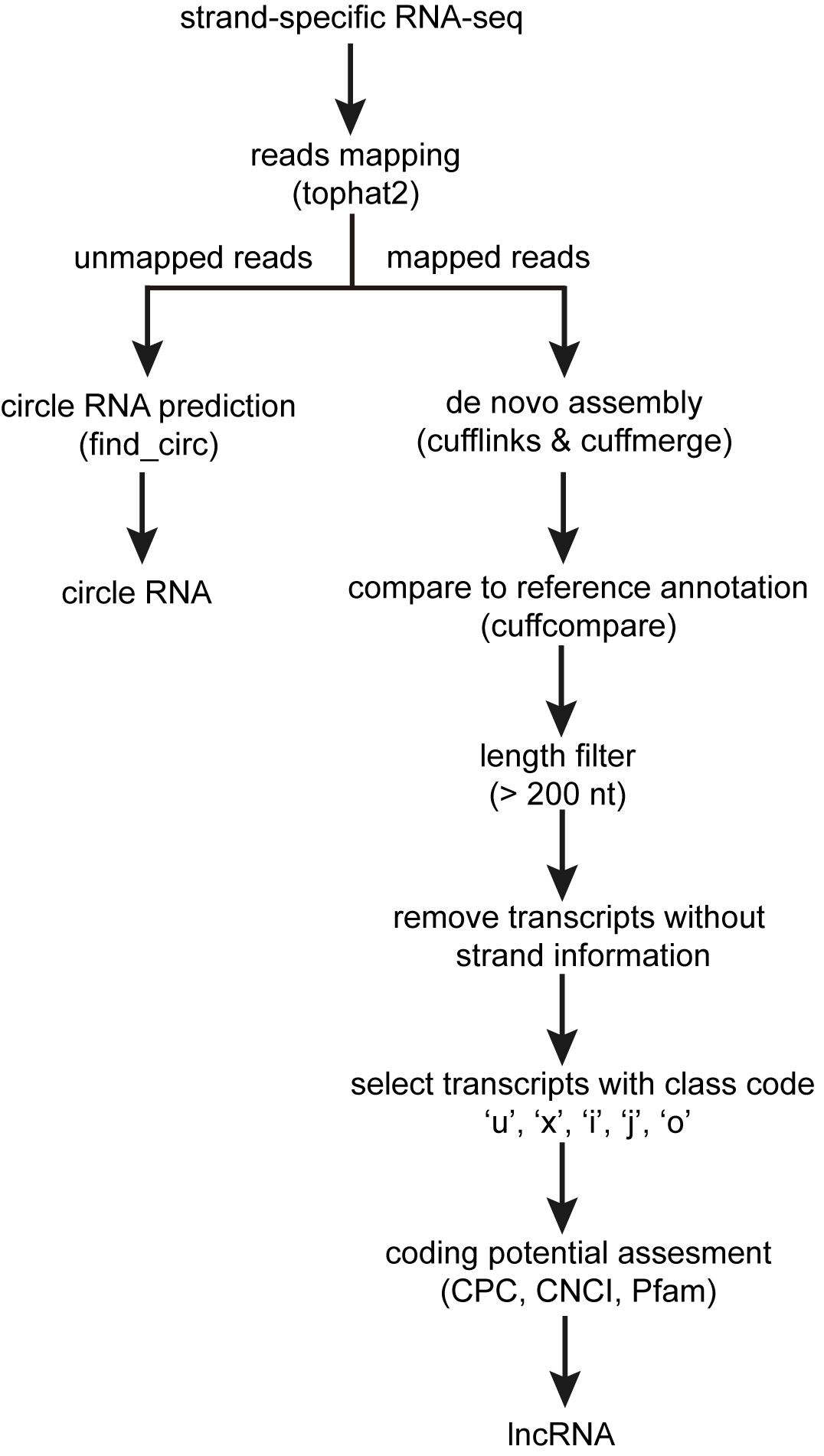


**Supplementary Figure S1.** An integrative computational pipeline for the systematic identification of lncRNAs and circRNA in tomato.


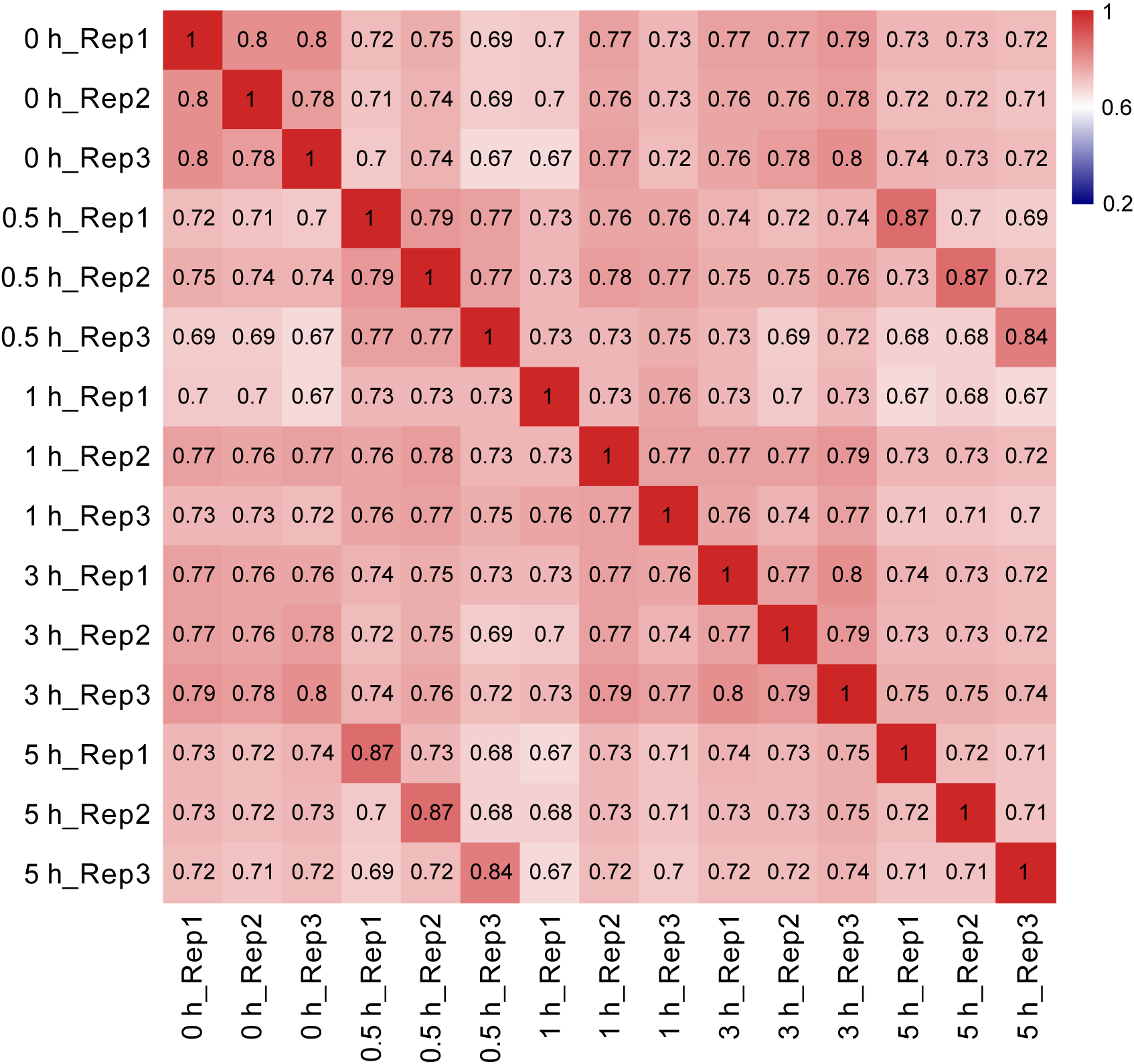


**Supplementary Figure S2.** SCC analysis of the RNA-seq data for the 15 samples using log2-transformed expression values.


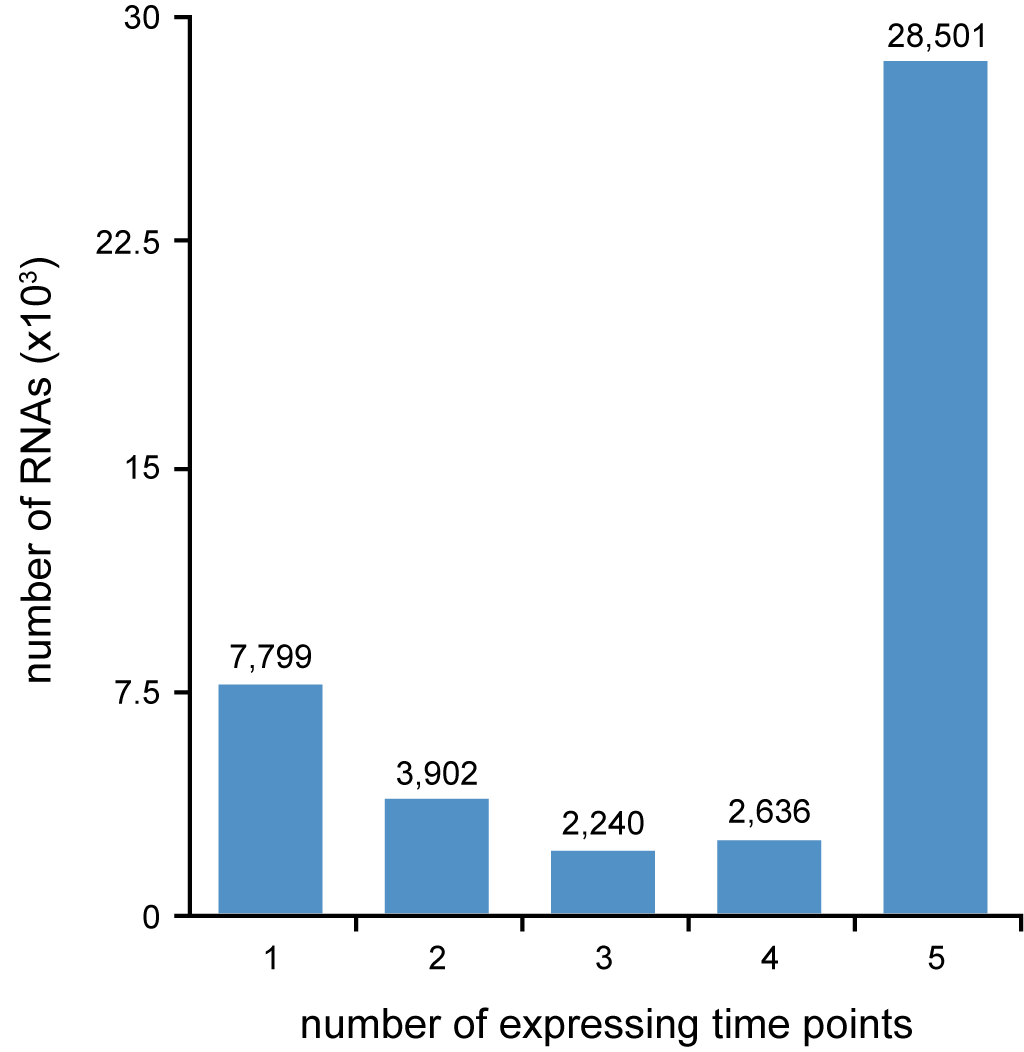


**Supplementary Figure S3.** Number of non-coding RNAs and mRNAs expressed in at least one time points.


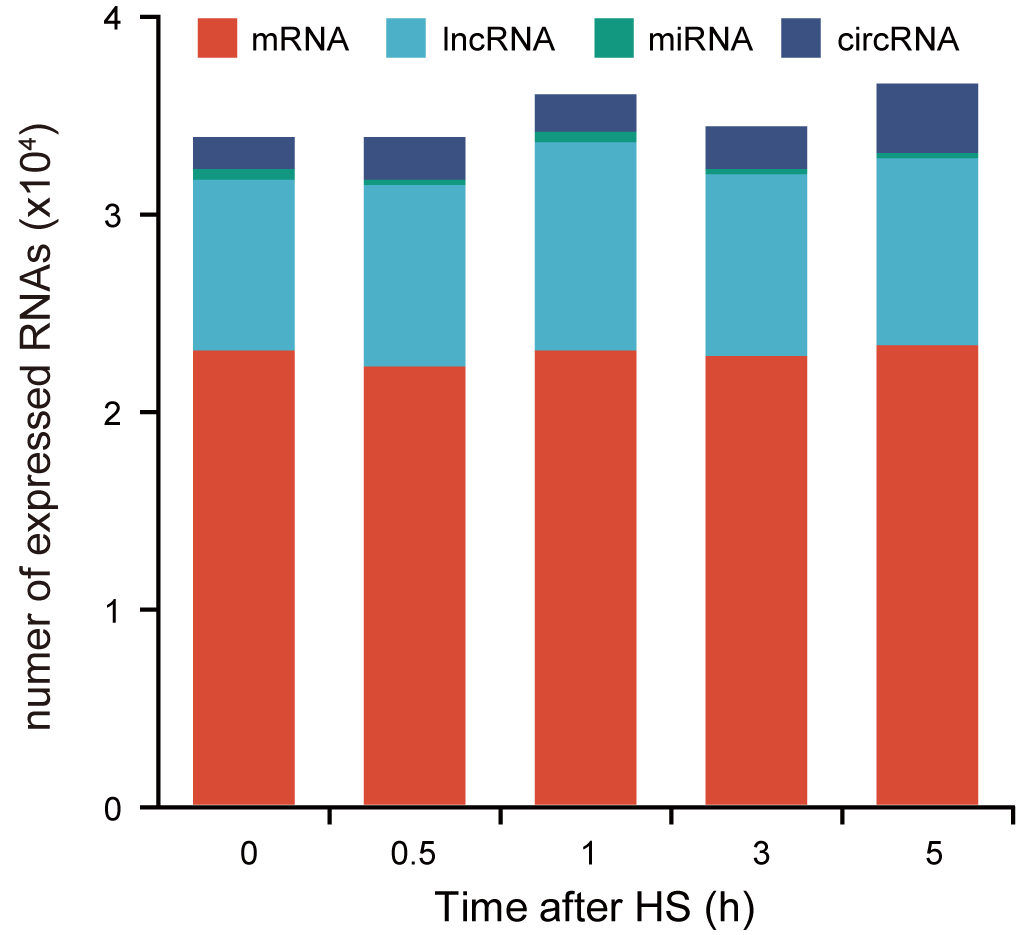


**Supplementary Figure S4.** Number of non-coding RNAs and mRNAs expressed in different time point.


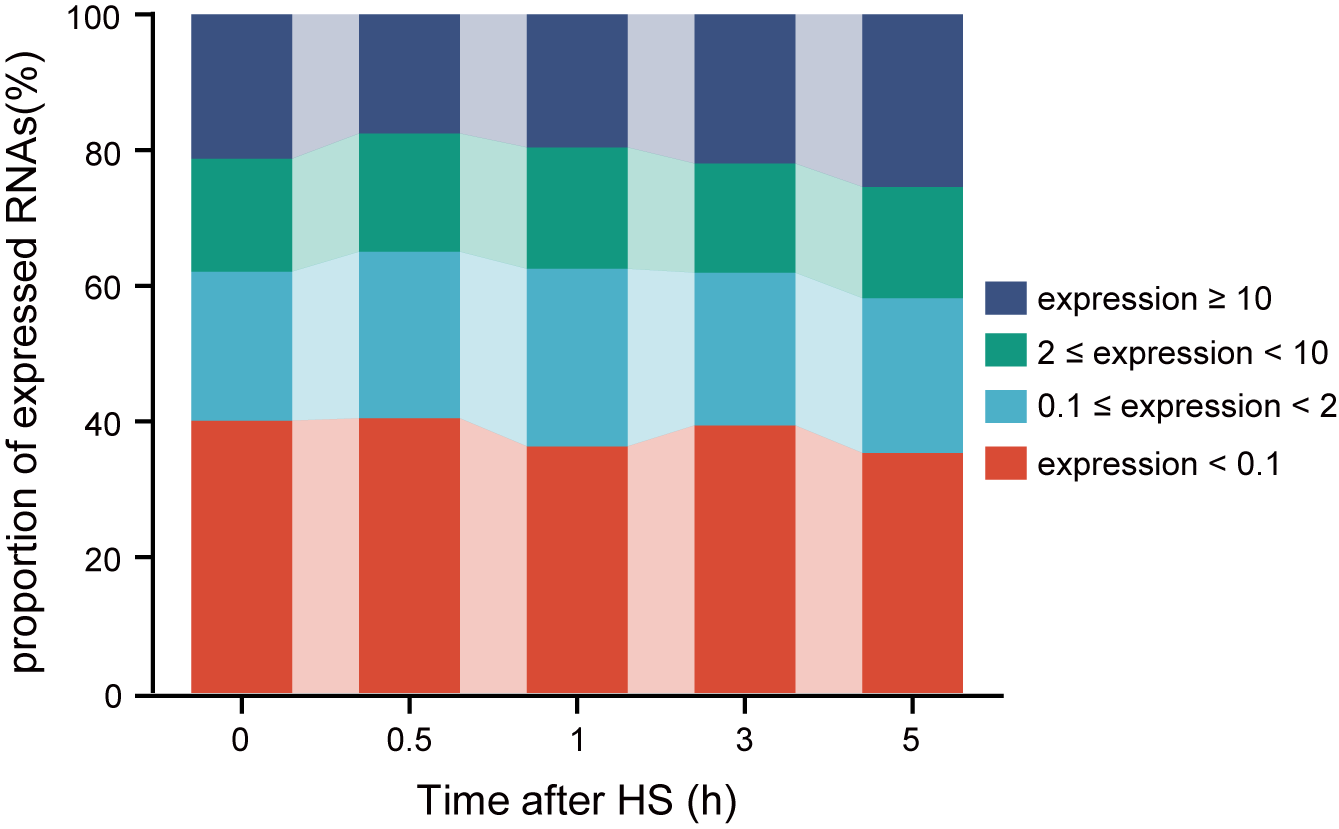


**Supplementary Figure S5.** Proportions of non-coding RNAs and mRNAs expressed in different time points.


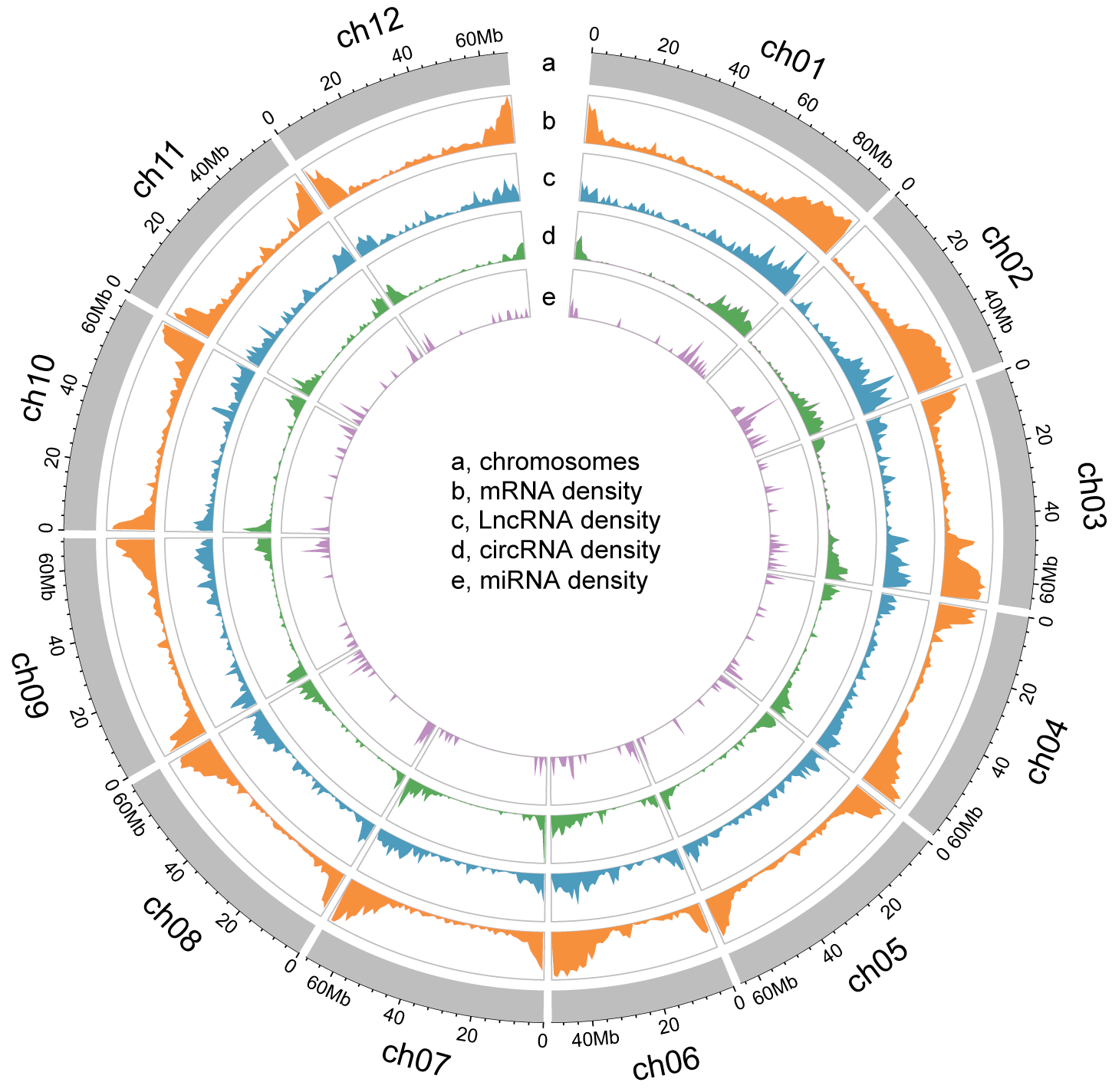


**Supplementary Figure S6.** The density of non-coding RNAs and mRNAs along the 12 chromosomes.


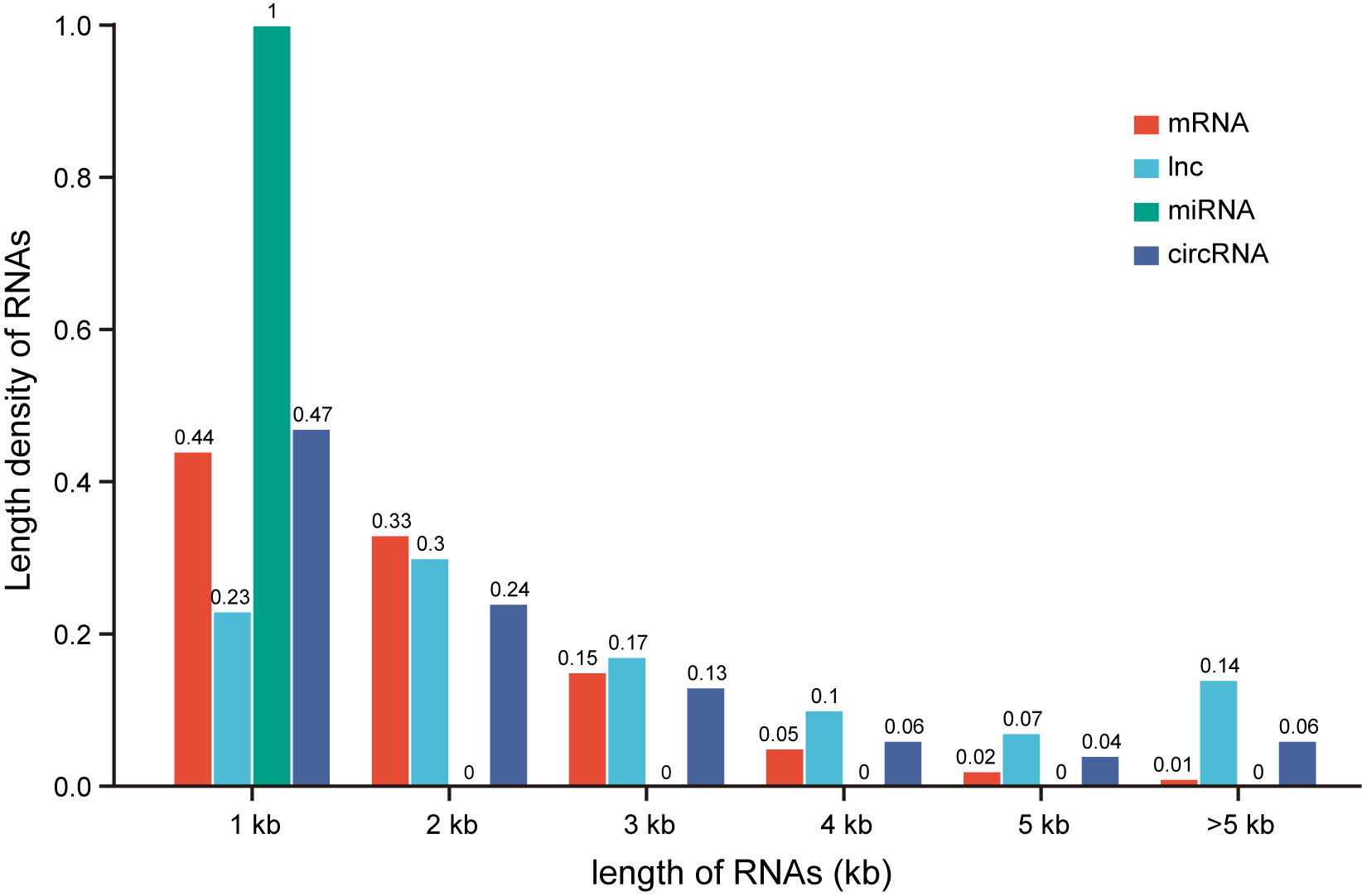


**Supplementary Figure S7.** Length distributions of non-coding RNAs and mRNAs.


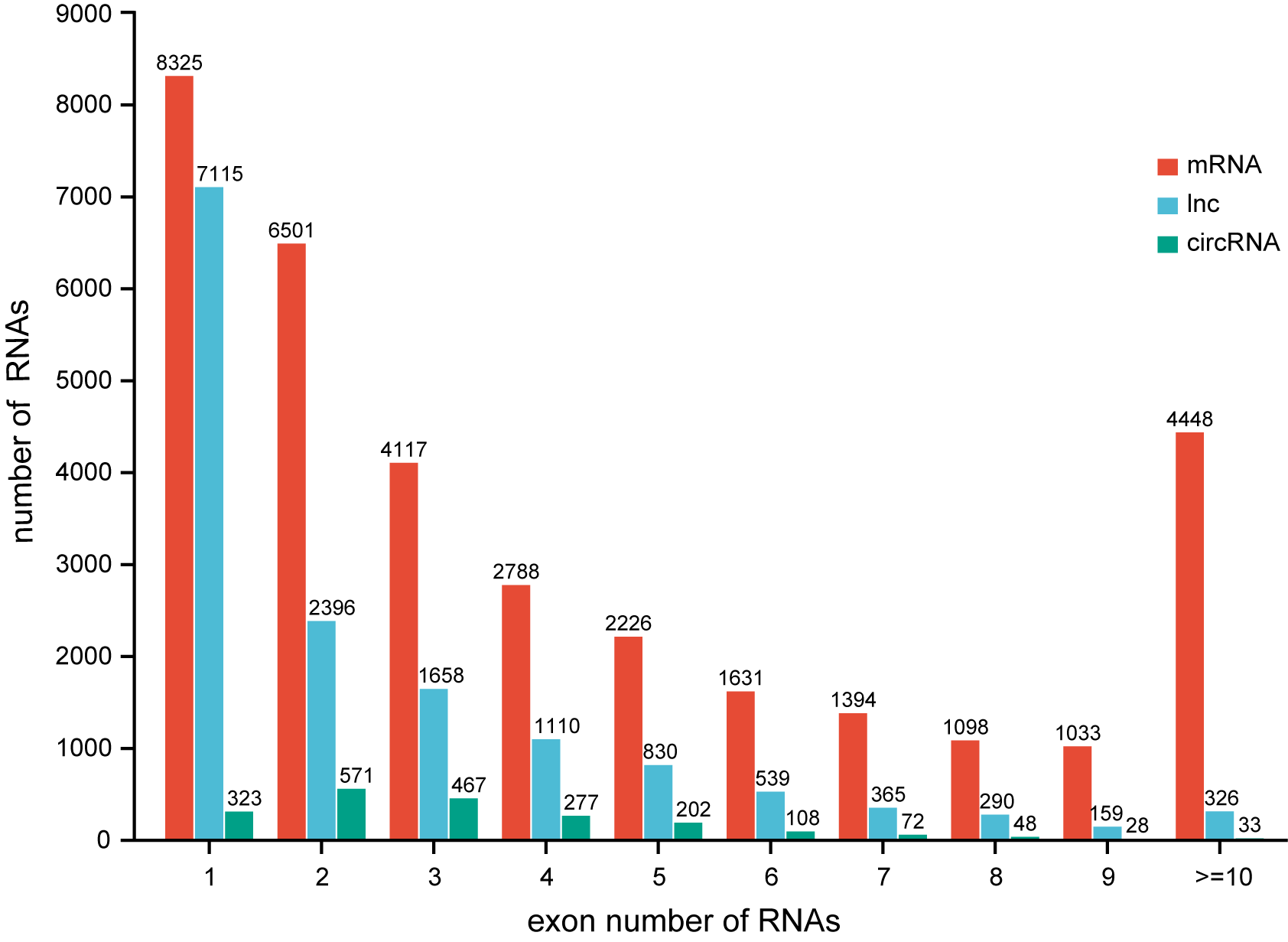


**Supplementary Figure S8.** Exon number of non-coding RNAs and mRNAs.


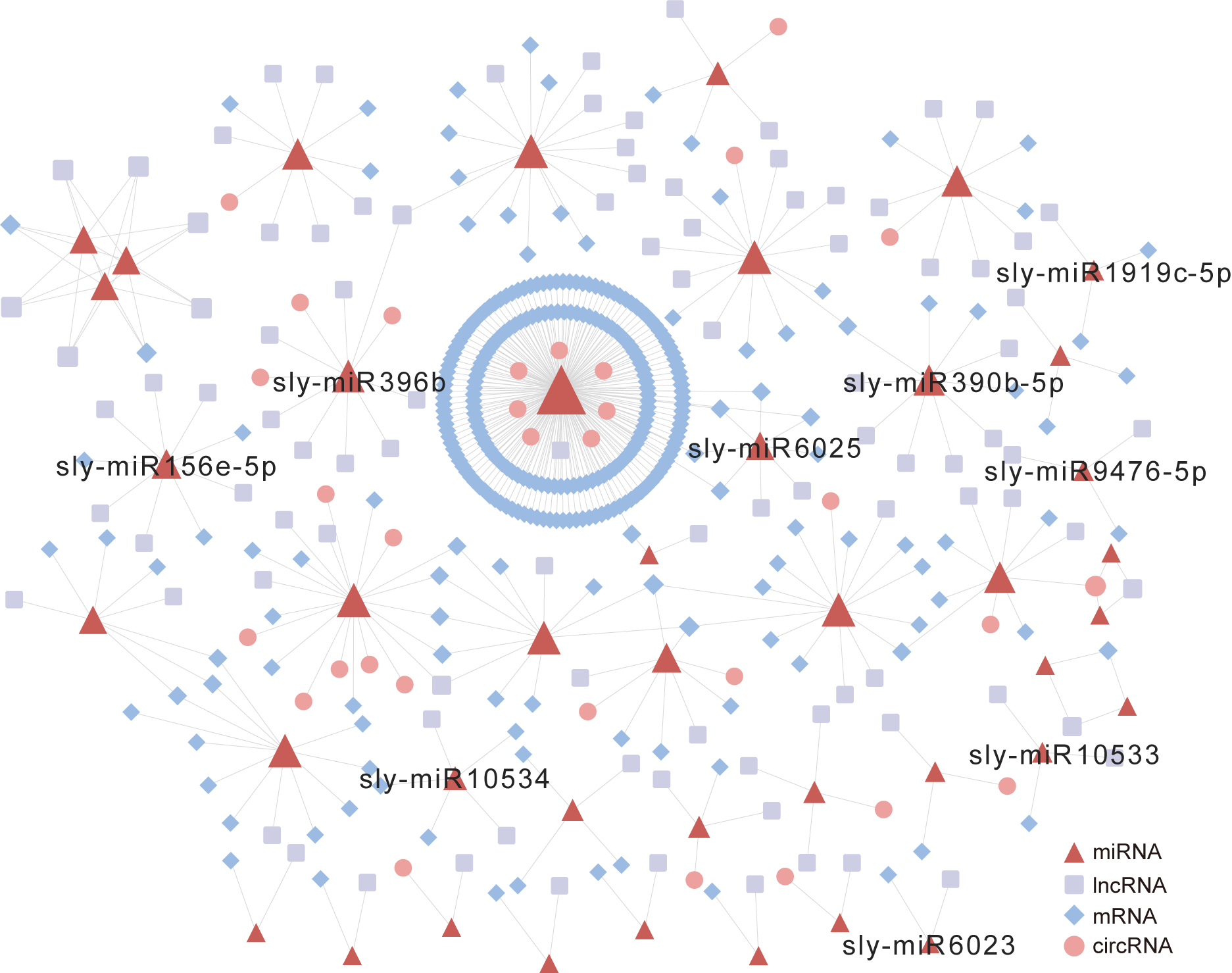


**Supplementary Figure S9.** The ceRNA regulatory networks under HS.


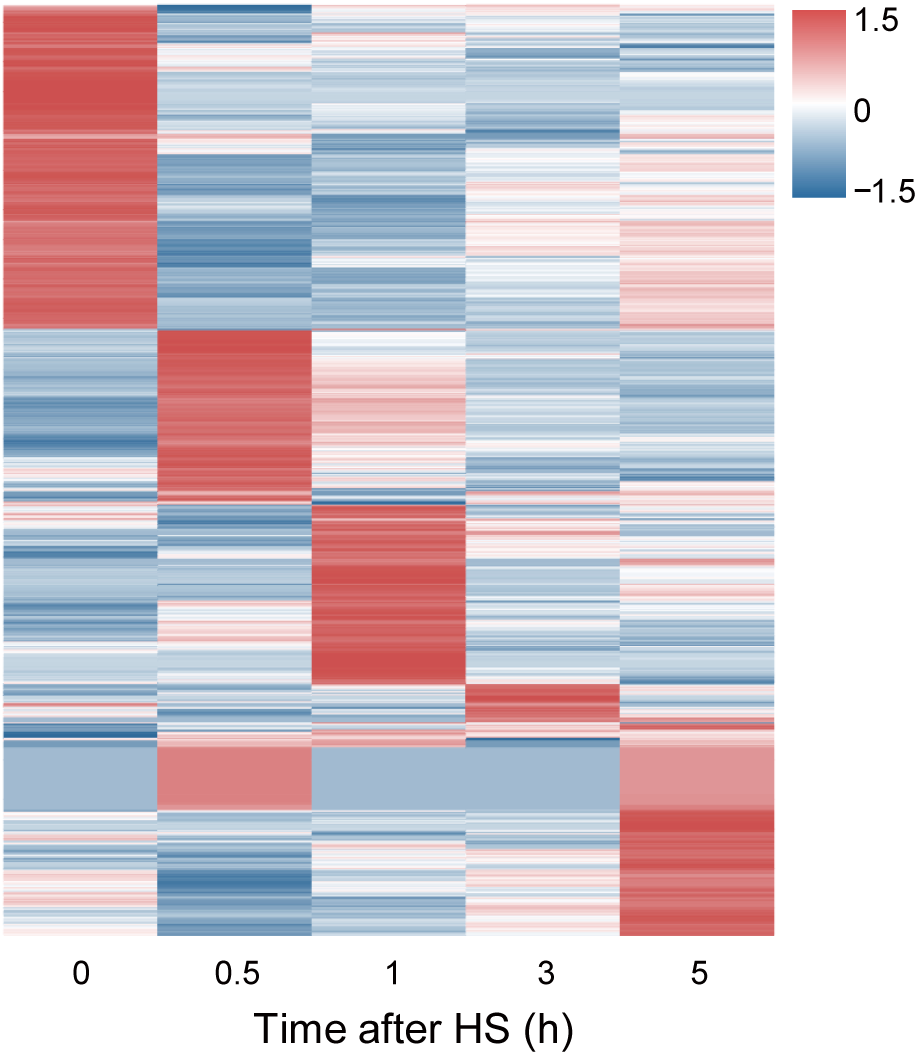


**Supplementary Figure S10.** The heatmap of expression patterns of 3,438 time-specific non-coding RNAs and 3,411 time-specific mRNAs.


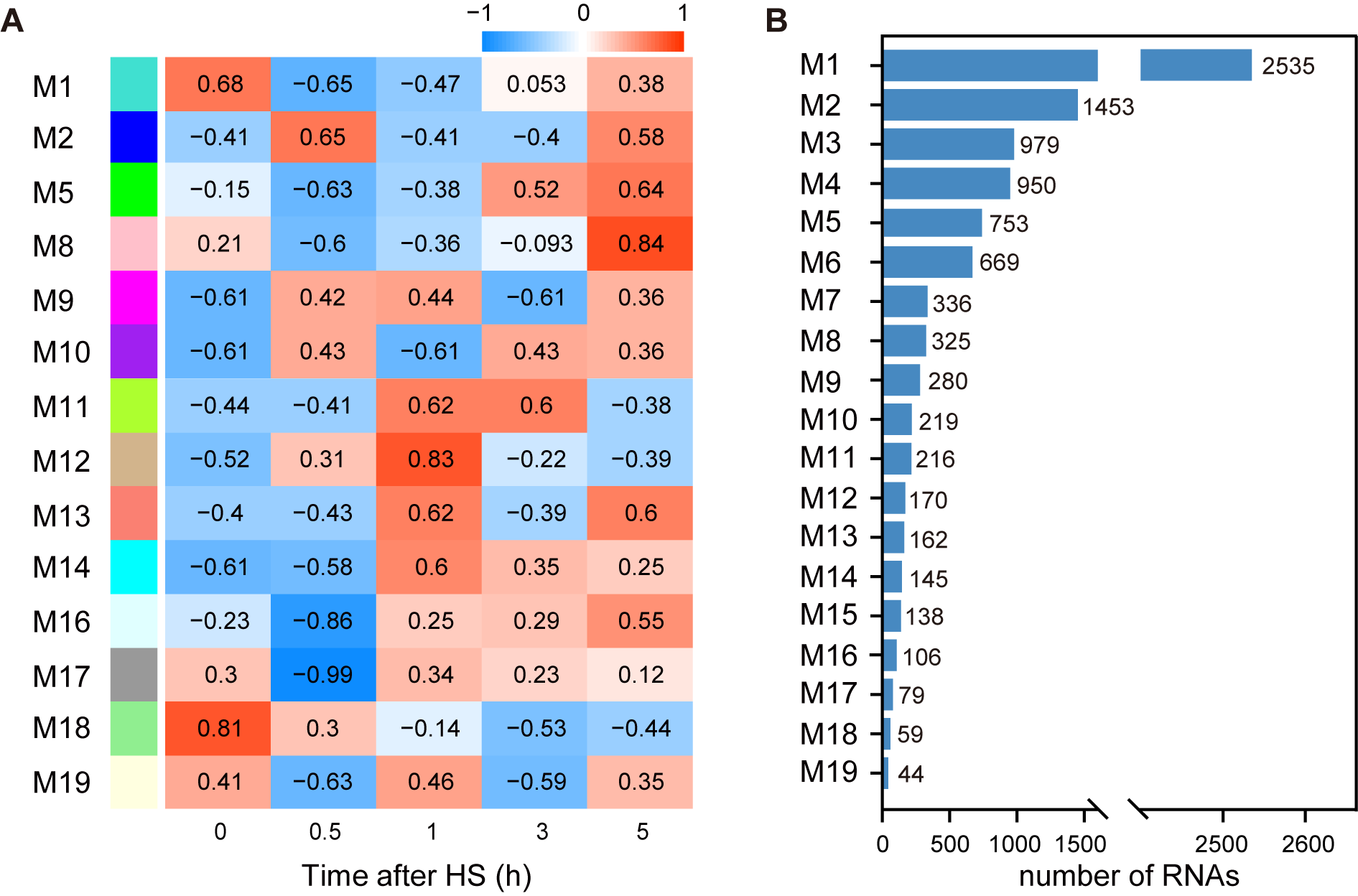


**Supplementary Figure S11.** **Co-expression modules detected using WGCNA.** (A) The correlation coefficients of each module with each time points. (B) Number of RNAs in each co-expression modules.


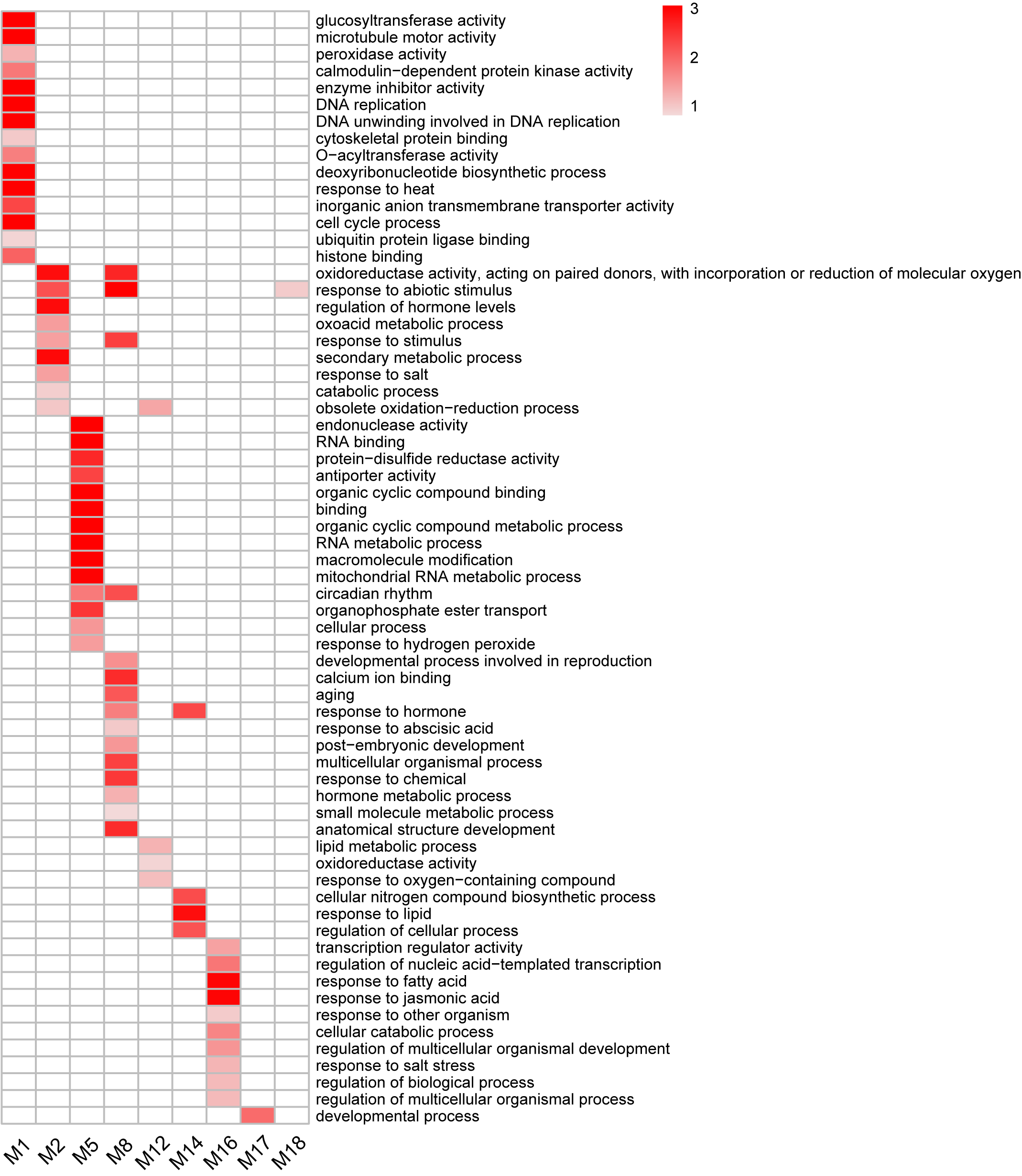


**Supplementary Figure S12.** Function enrichment analysis of each co-expression module. No enriched GO terms were identified in M9, M10, M11, M13 and M19.


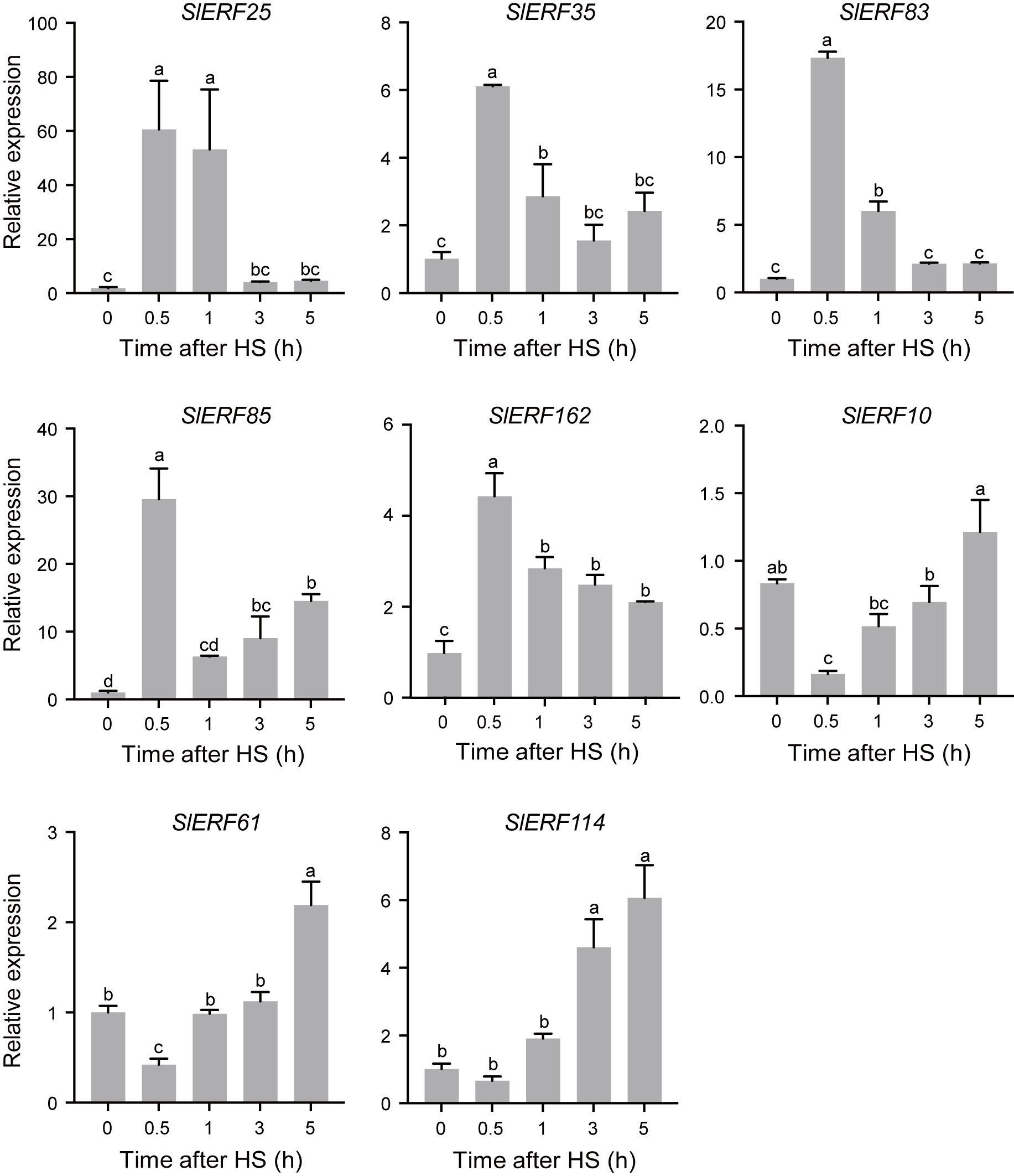


**Supplementary Figure S13.** Expression models of eight *ERFs* in tomato buds under HS using RT-qPCR. Values are mean ± SD (n = 3). Different letters represent significant differences at *P* < 0.05 (one-way ANOVA and Tukey’s multiple comparisons test).


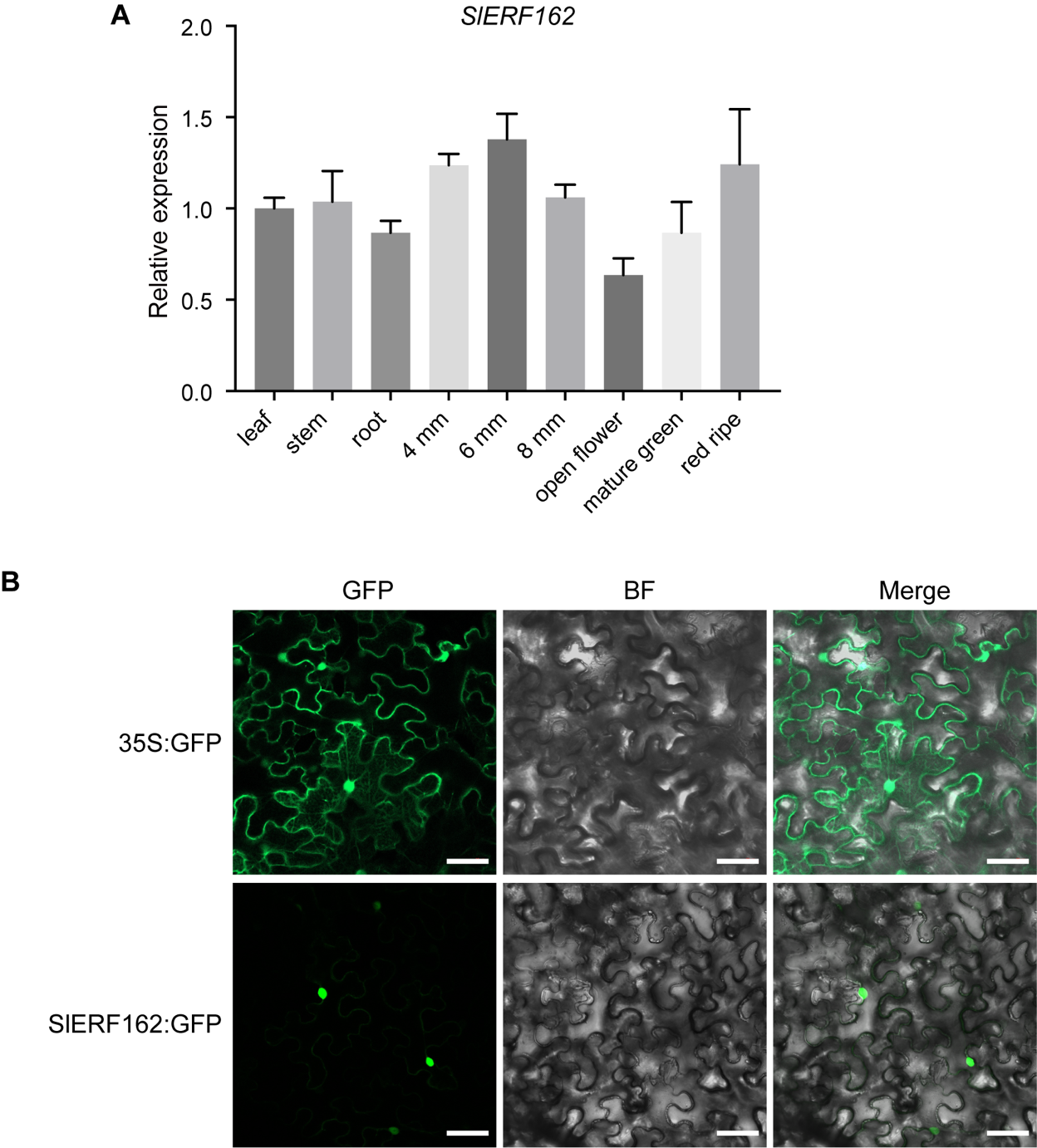


**Supplementary Figure S14. Tissue expression and subcellular localization of SlERF162.** (A) Expression levels of SlERF162 in different tomato tissues using RT-qPCR. Values are mean ± SD (n = 3). (B) Subcellular localization of SlERF162. Bars = 50 μm. BF, bright field. GFP, green fluorescent protein.


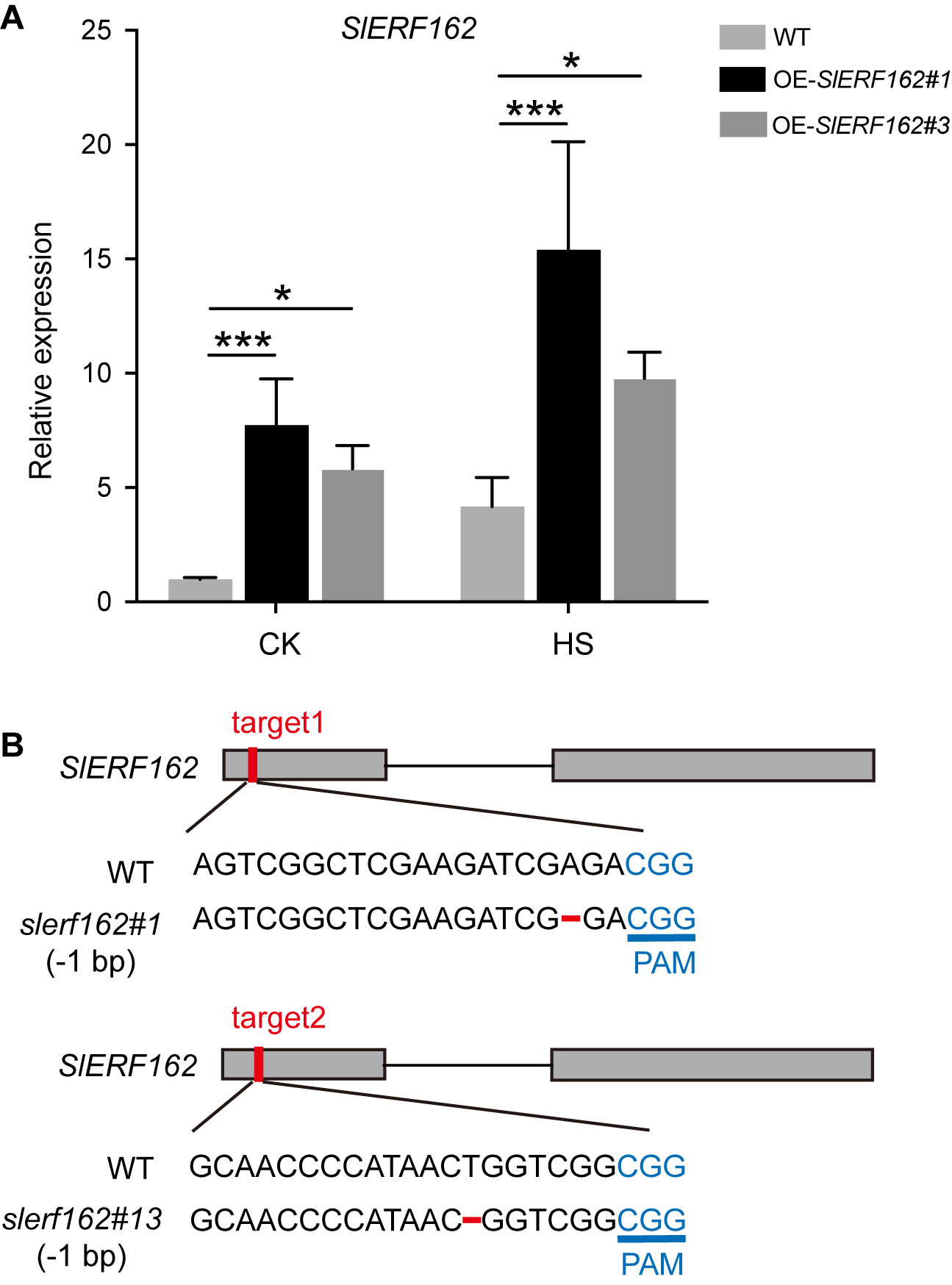


**Supplementary Figure S15.** **Obtaining *SlERF162* overexpression and mutant plants.** (A) Expression levels of *SlERF162* in the WT and OE-*SlERF162* lines under CK and HS conditions using RT-qPCR. Values are mean ± SD (n = 3). Asterisks indicate the significant differences (**P* < 0.05, ****P* < 0.001, two-way ANOVA and Tukey’s multiple comparisons test). (B) Alleles in the CRISPR/Cas9-mediated *SlERF162* mutant lines. CK, control check. HS, heat stress. OE, overexpression. WT, wild type.

**
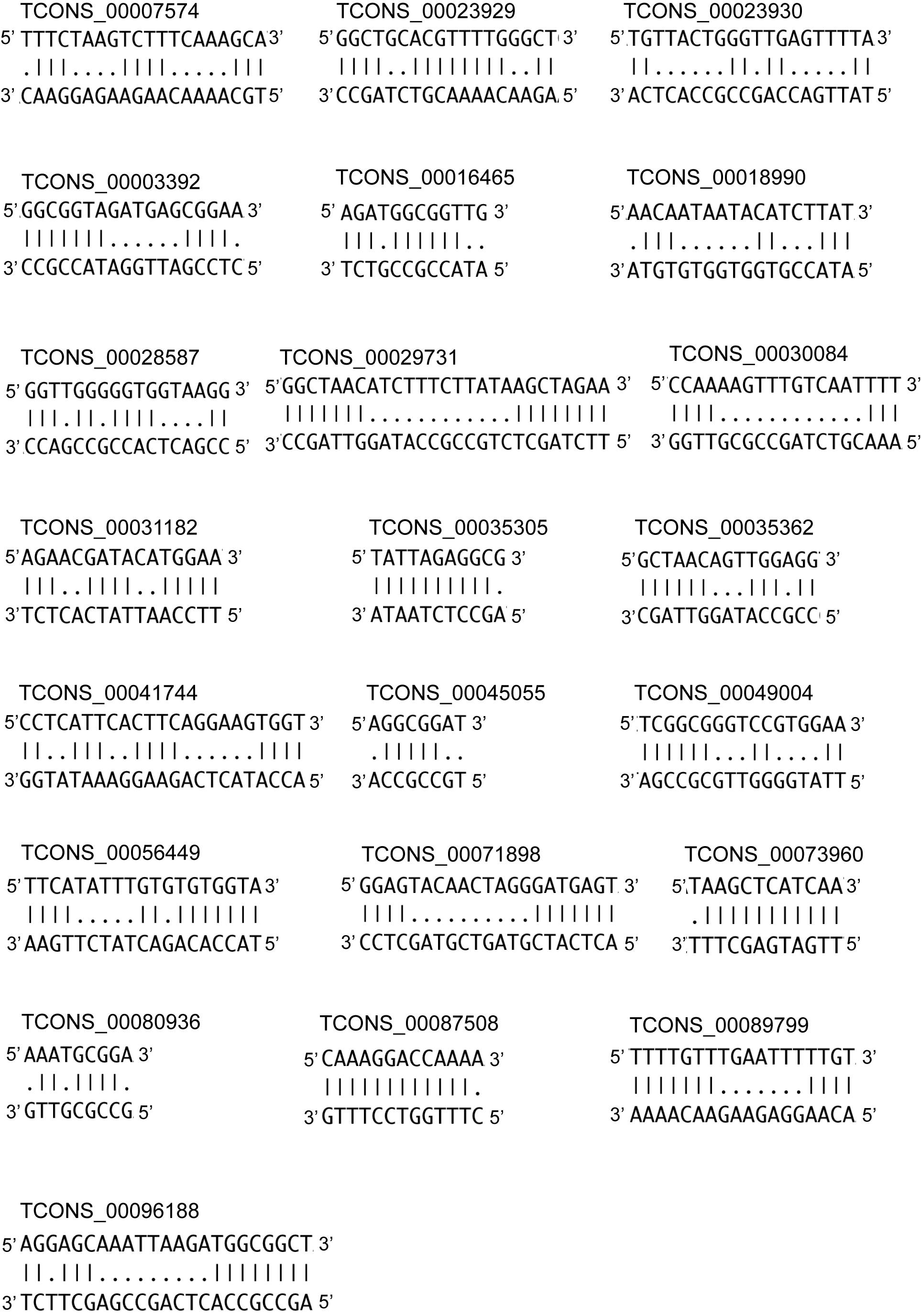
**

**Supplementary Figure S16.** The complementarity of sequences between SlERF162 and 22 lncRNAs.


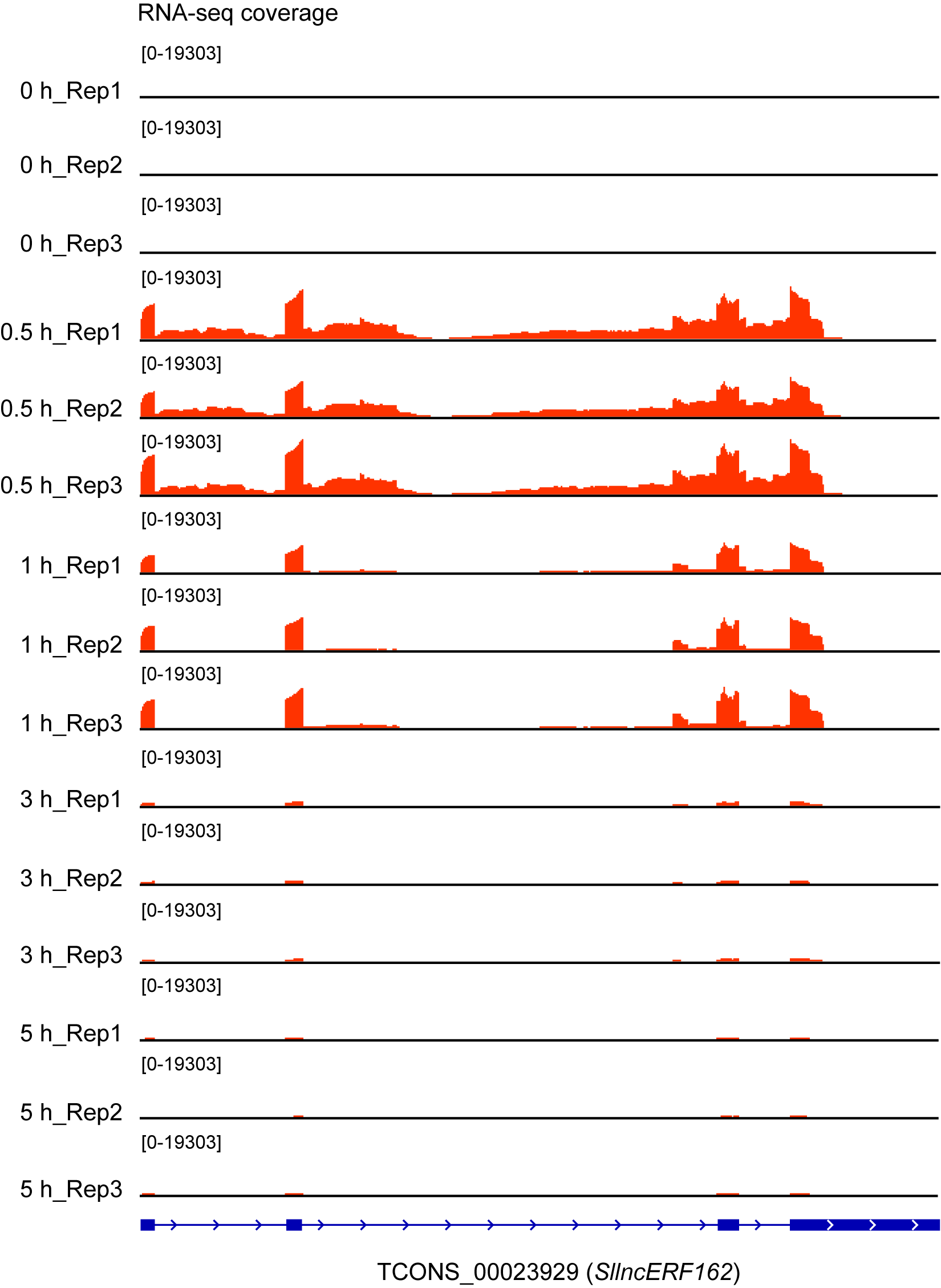


**Supplementary Figure S17.** The RNA-seq coverage of the TCONS_00023929 (*SllncERF162*) was presented under different time points.


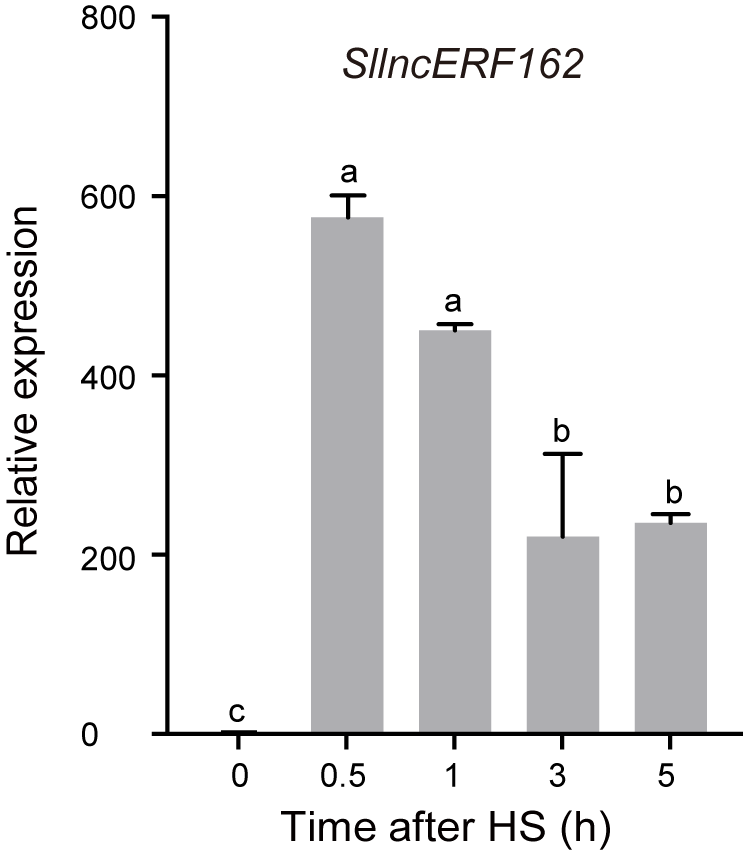


**Supplementary Figure S18.** Expression level of *SllncERF162* in tomato buds under HS using RT-qPCR. Values are mean ± SD (n = 3). Different letters represent significant differences at *P* < 0.05 (one-way ANOVA and Tukey’s multiple comparisons test).


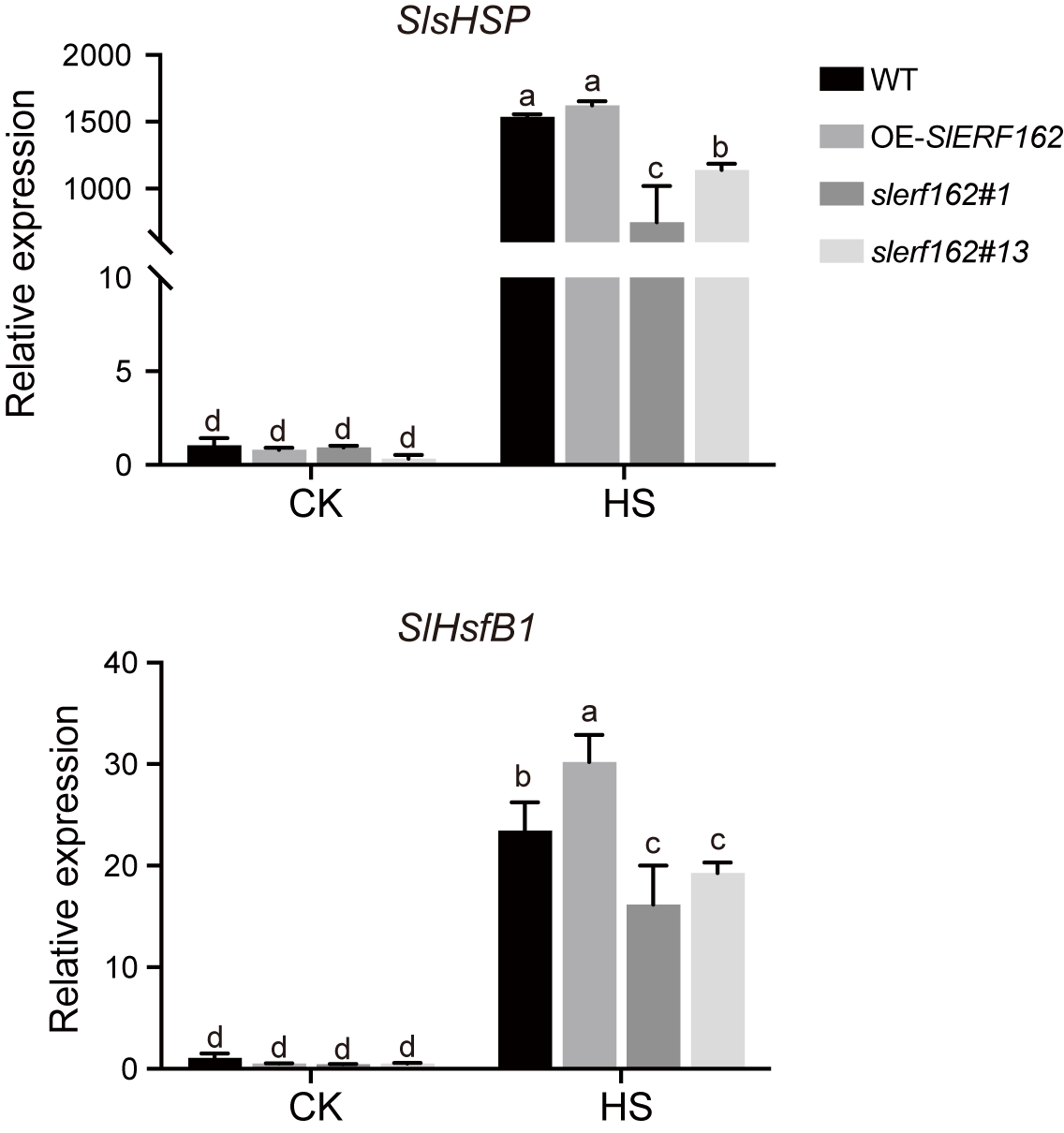


**Supplementary Figure S19.** Expression levels of *SlsHSP* and *SlHsfB1* in the WT, OE-*SlERF162#1*, and *slerf* lines under CK and HS using RT-qPCR. Values are mean ± SD (n = 3). Different letters represent significant differences at *P* < 0.05 (two-way ANOVA and Tukey’s multiple comparisons test). CK, control check. HS, heat stress. OE, overexpression. WT, wild type.


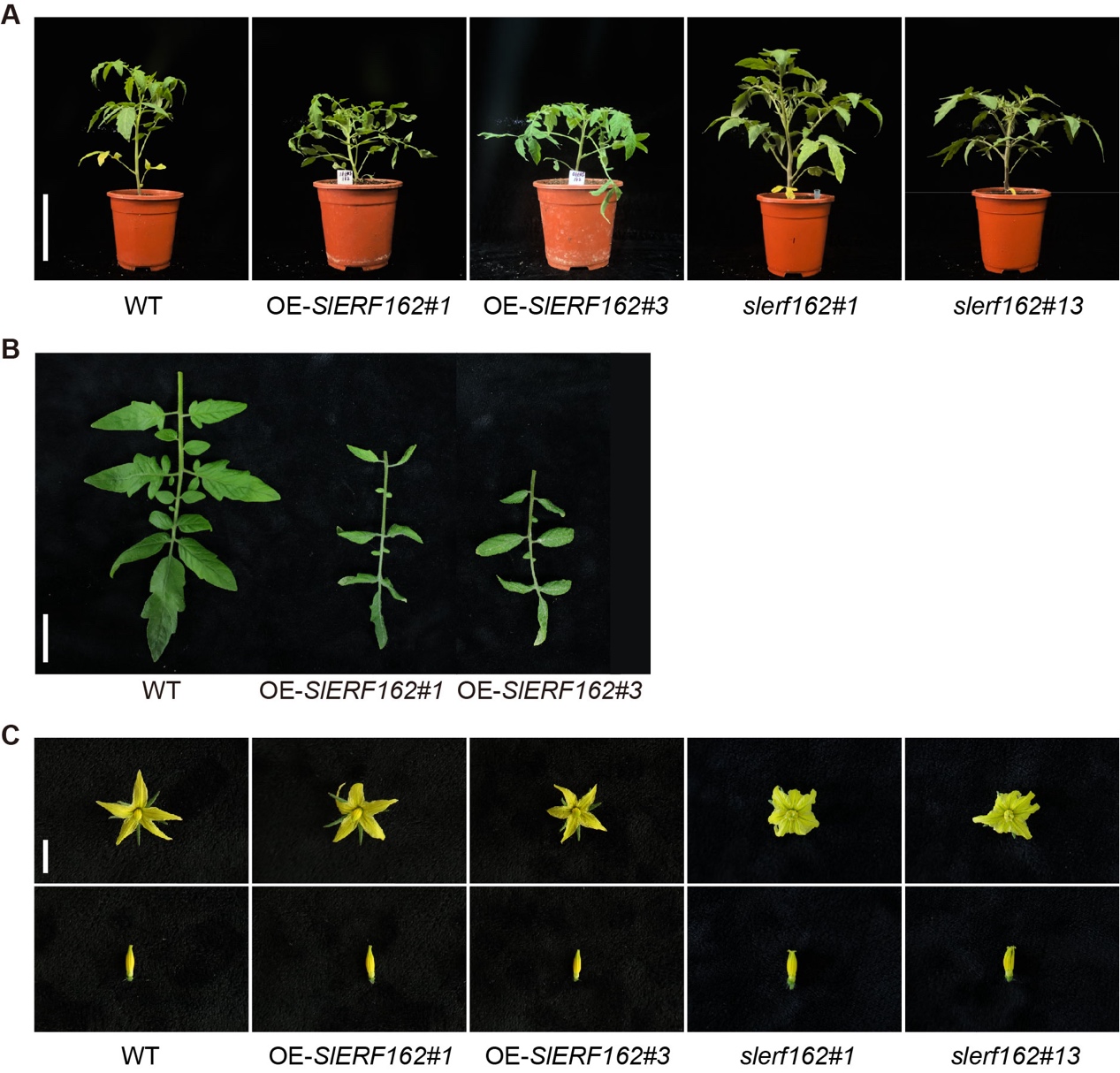


**Supplementary Figure S20.** The vegetative and reproductive growth phenotypes of SlERF162 transgenic plants. (A) The plant appearance of SlERF162 transgenic plants. Bars = 10 cm. (B) The leaf morphology of OE-*SlERF162* lines. Bars = 3 cm. (C) The flower and stamen morphology of SlERF162 transgenic plants. Bars = 1 cm.
