# Supplementary material for: Genome-wide expression atlas of tomato flower buds revealed the SllncERF162-SlERF162 module associated with basal thermotolerance
Source: Hortic Res. 2025 Jul 31;12(11):uhaf205. doi: 10.1093/hr/uhaf205 (PMC12574544; doi:10.1093/hr/uhaf205)
Supplement: Web_Material_uhaf205 [file web_material_uhaf205.zip › Supplementary Tables 1-6.docx]

**Supplementary Table S1**. Summary of strand-specific RNA-Seq data for fifteen libraries.

| **Sample** | **Raw reads** | **Clean reads** | **Raw Base (G)** | **Clean Base (G)** | **Effective Rate (%)** | **Error Rate (%)** | **Q20 (%)** | **Q30 (%)** | **GC Content (%)** | **overall read mapping rate (%)** |
| --- | --- | --- | --- | --- | --- | --- | --- | --- | --- | --- |
| 0h-1 | 73,785,579 | 73,313,568 | 22.14 | 21.99 | 99.36 | 0.03 | 97.79 | 93.58 | 41.34 | 93.50 |
| 0h-2 | 73,953,651 | 73,127,044 | 22.19 | 21.94 | 98.88 | 0.03 | 97.35 | 92.58 | 41.30 | 92.80 |
| 0h-3 | 66,540,096 | 65,902,941 | 19.96 | 19.77 | 99.04 | 0.03 | 97.68 | 93.25 | 41.25 | 93.70 |
| 0.5h-1 | 72,629,371 | 72,059,644 | 21.79 | 21.62 | 99.22 | 0.03 | 97.98 | 94.00 | 41.27 | 94.10 |
| 0.5h-2 | 71,036,322 | 70,457,558 | 21.31 | 21.14 | 99.19 | 0.03 | 97.76 | 93.47 | 41.39 | 94.10 |
| 0.5h-3 | 74,343,262 | 73,851,284 | 22.30 | 22.16 | 99.34 | 0.02 | 98.55 | 95.36 | 41.39 | 95.10 |
| 1h-1 | 72,709,633 | 72,143,034 | 21.81 | 21.64 | 99.22 | 0.03 | 97.75 | 93.50 | 41.01 | 93.80 |
| 1h-2 | 76,351,797 | 75,942,073 | 22.91 | 22.78 | 99.46 | 0.03 | 97.51 | 92.93 | 41.11 | 93.20 |
| 1h-3 | 68,837,192 | 68,351,831 | 20.65 | 20.51 | 99.29 | 0.03 | 97.72 | 93.43 | 41.09 | 93.60 |
| 3h-1 | 78,850,586 | 78,306,325 | 23.66 | 23.49 | 99.31 | 0.03 | 97.81 | 93.62 | 41.08 | 93.90 |
| 3h-2 | 72,592,793 | 72,018,484 | 21.78 | 21.61 | 99.21 | 0.03 | 97.72 | 93.40 | 41.06 | 93.60 |
| 3h-3 | 67,824,057 | 67,454,551 | 20.35 | 20.24 | 99.46 | 0.03 | 97.74 | 93.44 | 41.17 | 93.70 |
| 5h-1 | 77,525,357 | 77,091,111 | 23.26 | 23.13 | 99.44 | 0.03 | 97.73 | 93.45 | 41.23 | 93.20 |
| 5h-2 | 76,592,012 | 76,067,389 | 22.98 | 22.82 | 99.32 | 0.03 | 97.52 | 92.96 | 40.96 | 93.40 |
| 5h-3 | 72,609,242 | 72,108,119 | 21.78 | 21.63 | 99.31 | 0.03 | 97.67 | 93.41 | 41.05 | 93.50 |

**Supplementary Table S2**. Summary of smallRNA-seq data for fifteen libraries.

| **Sample** | **Raw reads** | **Clean reads** | **18~30nt reads** | **distinct reads** | **Rfam annotation** | | | | **unannotated reads** | **mapped reads** | **overall read mapping rate (%)** |
| --- | --- | --- | --- | --- | --- | --- | --- | --- | --- | --- | --- |
|  |  |  |  |  | **tRNA** | **rRNA** | **sRNA** | **snRNA** |  |  |  |
| 0h-1 | 27,463,127 | 26,991,314 | 24,336,703 | 4,364,307 | 22,888 | 215,883 | 14,271 | 38,997 | 4,080,143 | 3,119,183 | 76.45% |
| 0h-2 | 27,154,628 | 26,817,878 | 22,735,266 | 4,023,327 | 21,496 | 216,641 | 13,201 | 37,036 | 3,742,688 | 2,864,680 | 76.54% |
| 0h-3 | 26,068,285 | 25,719,950 | 21,852,391 | 4,512,025 | 24,631 | 194,852 | 15,221 | 41,432 | 4,244,084 | 3,227,598 | 76.05% |
| 0.5h-1 | 24,055,012 | 23,726,452 | 18,864,483 | 2,916,096 | 18,018 | 214,478 | 9,758 | 27,954 | 2,653,103 | 2,023,167 | 76.26% |
| 0.5h-2 | 22,736,487 | 22,357,624 | 16,848,713 | 2,402,119 | 13,484 | 198,373 | 8,063 | 23,461 | 2,164,953 | 1,659,624 | 76.66% |
| 0.5h-3 | 27,156,612 | 26,720,545 | 21,094,271 | 3,209,058 | 19,954 | 212,675 | 10,665 | 32,777 | 2,940,956 | 2,238,213 | 76.10% |
| 1h-1 | 26,519,805 | 26,096,969 | 20,063,687 | 3,347,657 | 18,965 | 211,115 | 11,117 | 34,147 | 3,079,988 | 2,331,809 | 75.71% |
| 1h-2 | 30,734,704 | 29,906,239 | 20,612,922 | 3,558,618 | 19,783 | 172,562 | 11,152 | 33,798 | 3,327,545 | 2,511,321 | 75.47% |
| 1h-3 | 24,783,377 | 24,311,139 | 17,574,488 | 3,390,918 | 18,232 | 178,886 | 10,859 | 33,052 | 3,156,530 | 2,408,303 | 76.30% |
| 3h-1 | 23,280,856 | 22,887,424 | 18,514,378 | 3,446,345 | 18,971 | 203,297 | 11,387 | 33,644 | 3,186,407 | 2,417,918 | 75.88% |
| 3h-2 | 24,561,607 | 24,029,199 | 18,129,505 | 3,715,624 | 18,531 | 186,716 | 12,207 | 34,754 | 3,470,138 | 2,648,338 | 76.32% |
| 3h-3 | 24,148,849 | 23,613,397 | 18,111,314 | 3,804,538 | 20,125 | 169,225 | 12,115 | 35,598 | 3,573,897 | 2,714,850 | 75.96% |
| 5h-1 | 21,783,377 | 21,435,622 | 18,115,972 | 4,125,848 | 20,928 | 173,915 | 13,676 | 39,999 | 3,884,254 | 2,938,390 | 75.65% |
| 5h-2 | 31,790,269 | 31,354,489 | 26,405,201 | 4,850,856 | 25,167 | 239,517 | 16,661 | 47,523 | 4,531,024 | 3,374,926 | 74.48% |
| 5h-3 | 22,158,344 | 21,737,834 | 17,011,049 | 4,160,930 | 21,359 | 163,976 | 13,354 | 37,605 | 3,931,301 | 2,991,125 | 76.08% |

**Supplementary Table S3**. Number of differently expressed RNAs.

| **Time point** | **mRNA** | | | **LncRNA** | | | **circRNA** | | | **miRNA** | | |
| --- | --- | --- | --- | --- | --- | --- | --- | --- | --- | --- | --- | --- |
|  | **up** | **down** | **total** | **up** | **down** | **total** | **up** | **down** | **total** | **up** | **down** | **total** |
| 0h_vs_0.5h | 1,892 | 3,120 | 5,012 | 1,999 | 1,709 | 3,708 | 36 | 2 | 38 | 7 | 8 | 15 |
| 0h_vs_1h | 2,114 | 2,383 | 4,497 | 1,815 | 1,237 | 3,052 | 21 | 2 | 23 | 5 | 3 | 8 |
| 0h_vs_3h | 844 | 1,823 | 2,667 | 1,039 | 1,101 | 2,140 | 7 | 4 | 11 | 1 | 9 | 10 |
| 0h_vs_5h | 1,150 | 1,225 | 2,375 | 952 | 1,054 | 2,006 | 44 | 1 | 45 | 1 | 6 | 7 |

**Supplementary Table S4**. Number of RNAs in time-specific and module-specific.

| **Time point/module** | **time-specific** | **module-specific** | **common** | **Ratio (%)** |
| --- | --- | --- | --- | --- |
| 0h-brown | 2,410 | 979 | 798 | 81.51% |
| 0.5h-yellow | 1,697 | 950 | 601 | 63.26% |
| 1h-red | 1,378 | 669 | 574 | 85.80% |
| 3h-midnightblue | 334 | 138 | 71 | 51.45% |
| 5h-black | 1,030 | 336 | 274 | 81.55% |

**Supplementary Table S5**. The TFs in five modules.

| **gene** | **TF family** | **module** |
| --- | --- | --- |
| SlT01G016150 | bHLH | brown |
| SlT01G038000 | MYB-related | brown |
| SlT02G017540 | MYB-related | brown |
| SlT02G023500 | MYB-related | brown |
| SlT02G024790 | C3H | brown |
| SlT02G026170 | HD-ZIP | brown |
| SlT03G021250 | MADS-MIKC | brown |
| SlT04G016820 | WRKY | brown |
| SlT04G026710 | E2F-DP | brown |
| SlT05G002240 | bHLH | brown |
| SlT05G003590 | C3H | brown |
| SlT05G009700 | SBP | brown |
| SlT06G006310 | B3 | brown |
| SlT07G013700 | NAC | brown |
| SlT08G021490 | GRAS | brown |
| SlT09G023110 | MYB | brown |
| SlT11G021390 | MYB | brown |
| SlT12G019510 | MADS-MIKC | brown |
| SlT01G002360 | OFP | yellow |
| SlT01G022730 | ERF | yellow |
| SlT01G028080 | MADS-M-type | yellow |
| SlT01G028660 | bHLH | yellow |
| SlT01G031800 | zf-HD | yellow |
| SlT02G008010 | GATA | yellow |
| SlT02G014900 | HSF | yellow |
| SlT02G028920 | ERF | yellow |
| SlT02G028970 | HSF | yellow |
| SlT03G004230 | ERF | yellow |
| SlT03G018770 | EIL | yellow |
| SlT03G019230 | HSF | yellow |
| SlT04G005750 | bHLH | yellow |
| SlT04G005910 | ERF | yellow |
| SlT04G006710 | GATA | yellow |
| SlT04G018570 | Dof | yellow |
| SlT04G021370 | bHLH | yellow |
| SlT05G002660 | MYB | yellow |
| SlT05G002720 | NAC | yellow |
| SlT05G002810 | MYB | yellow |
| SlT05G019960 | ERF | yellow |
| SlT06G009040 | ERF | yellow |
| SlT06G010940 | MYB | yellow |
| SlT06G020920 | OFP | yellow |
| SlT06G026630 | NAC | yellow |
| SlT06G026680 | MYB | yellow |
| SlT07G011570 | GRF | yellow |
| SlT07G020770 | BES1 | yellow |
| SlT07G021330 | NAC | yellow |
| SlT07G021470 | SBP | yellow |
| SlT08G011660 | bHLH | yellow |
| SlT08G011830 | HSF | yellow |
| SlT08G019550 | ERF | yellow |
| SlT08G021620 | HSF | yellow |
| SlT09G002810 | HD-ZIP | yellow |
| SlT09G004870 | MYB | yellow |
| SlT09G017050 | HSF | yellow |
| SlT09G018890 | TUB | yellow |
| SlT09G019870 | GATA | yellow |
| SlT10G003820 | TCP | yellow |
| SlT10G017110 | OFP | yellow |
| SlT11G012830 | LOB | yellow |
| SlT11G020040 | MYB | yellow |
| SlT11G022500 | Dof | yellow |
| SlT12G002750 | C3H | yellow |
| SlT12G004410 | DBP | yellow |
| SlT12G017470 | ERF | yellow |
| SlTUNG000190 | Dof | yellow |
| SlT02G016160 | ERF | red |
| SlT02G019440 | WRKY | red |
| SlT02G023930 | GRAS | red |
| SlT02G027690 | bZIP | red |
| SlT03G008010 | PLATZ | red |
| SlT03G025530 | C2H2 | red |
| SlT04G003630 | NAC | red |
| SlT04G019330 | ERF | red |
| SlT04G019760 | WRKY | red |
| SlT04G026370 | DBB | red |
| SlT05G022110 | C2H2 | red |
| SlT06G017570 | NAC | red |
| SlT07G002040 | NAC | red |
| SlT07G015670 | WRKY | red |
| SlT07G020750 | NAC | red |
| SlT08G018010 | MYB | red |
| SlT10G022420 | G2-like | red |
| SlT10G022520 | NAC | red |
| SlT11G017700 | NAC | red |
| SlT12G000690 | MYB | red |
| SlT12G018560 | ERF | red |
| SlT07G016400 | MADS-M-type | midnightblue |
| SlT01G009840 | NAC | black |
| SlT01G036680 | ERF | black |
| SlT02G007370 | NAC | black |
| SlT02G007380 | NAC | black |
| SlT02G026950 | C2H2 | black |
| SlT02G031450 | NAC | black |
| SlT03G006370 | OFP | black |
| SlT03G017130 | C2H2 | black |
| SlT03G024640 | NAC | black |
| SlT05G002820 | Dof | black |
| SlT05G007490 | MADS-M-type | black |
| SlT05G007550 | MYB-related | black |
| SlT06G006760 | ERF | black |
| SlT08G006380 | Tify | black |
| SlT08G009580 | NAC | black |
| SlT08G015820 | NAC | black |
| SlT10G000140 | MYB-related | black |
| SlT10G013760 | ERF | black |
| SlT10G023420 | MYB-related | black |
| SlT11G007260 | MYB-related | black |
| SlT11G008830 | MADS-M-type | black |
| SlT11G009750 | NAC | black |

**Supplementary Table S6**. The candidate lncRNAs target *SlERF162*.

| **lncRNA** | **mRNA** | **dG** | **ndG** |
| --- | --- | --- | --- |
| TCONS_00007574 | SlT12G017470 | -16.18 | -0.1086 |
| TCONS_00023929 | SlT12G017470 | -10.64 | -0.1157 |
| TCONS_00023930 | SlT12G017470 | -10.64 | -0.1157 |
| TCONS_00003392 | SlT12G017470 | -9.15 | -0.1525 |
| TCONS_00016465 | SlT12G017470 | -12.13 | -0.1304 |
| TCONS_00018990 | SlT12G017470 | -4.6 | -0.1022 |
| TCONS_00028587 | SlT12G017470 | -11.61 | -0.1683 |
| TCONS_00029731 | SlT12G017470 | -13.15 | -0.2989 |
| TCONS_00030084 | SlT12G017470 | -12.8 | -0.1123 |
| TCONS_00031182 | SlT12G017470 | -18.71 | -0.1011 |
| TCONS_00035305 | SlT12G017470 | -6.15 | -0.246 |
| TCONS_00035362 | SlT12G017470 | -13.92 | -0.1123 |
| TCONS_00041744 | SlT12G017470 | -14.8 | -0.1165 |
| TCONS_00045055 | SlT12G017470 | -6.76 | -0.1988 |
| TCONS_00049004 | SlT12G017470 | -18.2 | -0.1264 |
| TCONS_00056449 | SlT12G017470 | -16.19 | -0.1349 |
| TCONS_00071898 | SlT12G017470 | -13.13 | -0.1527 |
| TCONS_00073960 | SlT12G017470 | -8.91 | -0.81 |
| TCONS_00080936 | SlT12G017470 | -12 | -0.1165 |
| TCONS_00087508 | SlT12G017470 | -8.56 | -0.1189 |
| TCONS_00089799 | SlT12G017470 | -11.76 | -0.1109 |
| TCONS_00096188 | SlT12G017470 | -10.97 | -0.1503 |
